# Validation and reproducibility of a novel flavonoid food frequency dietary assessment tool (Flav-Q) against multiple 24-h recalls across 12 months

**DOI:** 10.1007/s00394-026-03997-7

**Published:** 2026-05-26

**Authors:** E. Lorzadeh, K. Charlton, T. A. McCaffrey, K. Weston-Green, M. Batterham, K. Kent

**Affiliations:** 1 School of Medical, Indigenous and Health Sciences, Faculty of Science, Medicine and Health, University of Wollongong, Northfields Ave, Wollongong, NSW 2522 Australia; 2 Faculty of Science, Medicine and Health, Molecular Horizons, University of Wollongong, Wollongong, NSW 2522 Australia; 3 Department of Nutrition, Dietetics and Food, School of Clinical Sciences, Monash University, Melbourne, VIC 3168 Australia; 4 School of Mathematics and Applied Statistics, Statistical Consulting Centre, University of Wollongong, Wollongong, NSW 2500 Australia; 5 School of Health Sciences, Western Sydney University, Campbelltown, NSW 2560 Australia

**Keywords:** Flavonoids, Dietary assessment, Flavonoid food frequency questionnaire, FLAV-Q

## Abstract

**Purpose:**

Measurement of dietary flavonoid intake requires a reliable tool. This study validated and evaluated the reproducibility of a 23-item shortened flavonoid food frequency questionnaire (FLAV-Q), derived from the 96-item Kent & Charlton Flavonoid specific-FFQ.

**Methods:**

The FLAV-Q was validated against an average of repeated 24-h dietary recalls using Intake-24 completed quarterly over the period of a year that were adjusted to represent habitual intake by applying the multiple source method (MSM) in 80 Australian adults. FLAV-Q validity was estimated using the Wilcoxon signed-rank test, Spearman’s correlation coefficient, Bland-Altman plots, and Cohen’s kappa (κ). Reproducibility was assessed by comparing the FLAV-Qs at four timepoints.

**Results:**

FLAV-Q overestimated total flavonoid intake against habitual intake (443.2 mg/day versus 234.4 mg/day, *p* < 0.001) and all subclasses except for flavanones. Moderate agreement was detected for total flavonoids (*r* = 0.66, *p* < 0.001; κ = 0.45, *p* < 0.001), flavan-3-ols (*r* = 0.72, *p* < 0.001; κ = 0.53, *p* < 0.001), flavonols (*r* = 0.55, *p* < 0.001; κ = 0.40, *p* < 0.001), flavanones (*r* = 0.50, *p* < 0.001; κ = 0.30, *p* = 0.007) and fair but non-significant agreement for anthocyanins (*r* = 0.38, *p* < 0.001; κ = 0.15, *p* = 0.18) and flavones (*r* = 0.34, *p* < 0.001; κ = 0.20, *p* = 0.07). Bland-Altman plots showed a large bias (Bland-Altman index: 7.5%) for total flavonoid intake. FLAV-Q demonstrated moderate reproducibility across timepoints with mean percentage differences for total flavonoid intake ranging from 22% to 37%. Bland-Altman plots indicated moderate to small bias for reproducibility (Bland-Altman index: 2.5–3.8%).

**Conclusions:**

FLAV-Q demonstrates moderate to low validity and reproducibility for total flavonoids and the subclasses. Further validation for absolute intake values is necessary to understand and address the overestimation.

**Supplementary Information:**

The online version contains supplementary material available at 10.1007/s00394-026-03997-7.

## Introduction

As a subclass of polyphenols, flavonoids represent non-nutrient constituents prevalent solely in plant-derived foods. Consequently, various plant-based dietary sources such as red wine, blueberries, and tea contain flavonoids [1]. The human diet encompasses a minimum of 300 distinct flavonoid compounds [2]. Nevertheless, the majority of overall flavonoid intake originates from a subset of fewer than 30 specific flavonoids that are commonly categorised into six significant subclasses: anthocyanins, flavan-3-ols, flavanones, flavones, flavonols and isoflavones [3].

Emerging, consistent epidemiological, clinical and animal research demonstrates that diets rich in flavonoids are beneficial for preventing several chronic diseases that significantly affect the Australian population, including cancer, heart disease, and diabetes [4–8]. However, health outcomes may vary according to intake of different flavonoids [9, 10], partly due to variations in the bioactivity of these compounds and their circulating metabolites [2]. For instance, flavones and flavan-3-ols have shown associations with reduced cardiovascular mortality [11], and protective effects have been observed in relation to the intake of flavonols and flavones concerning the risk of breast cancer [12]. Moreover, higher consumption of anthocyanins, flavones, and flavonols has been associated with a reduced likelihood of developing colorectal cancer [13].

Research on the associations between flavonoid intake and health outcomes in populations is often limited by inadequate dietary assessment methods and a lack of understanding of typical dietary intake patterns [14]. The limited availability of region-specific food composition data on flavonoids hinders comprehensive assessment of population-level consumption of total flavonoids and their subclasses [15, 16]. Earlier epidemiological evidence used a flavonoid food content database which was developed by the United States Department of Agriculture (USDA) [1, 17]. In the 2010s, the Phenol-Explorer Database was established, providing high quality data for the mean concentrations of an additional 502 polyphenols in over 450 foods [18]. With the growing availability of food composition data for flavonoids, quantification of population intakes in recent years has become more widespread and population-based estimates have been reported to range from 11 mg/d in Brazil [19] to 629 mg/d in Australia [20]. However, inconsistencies in the intake estimates across populations persist due to methodological differences in measuring dietary intake and the use of different flavonoid subclasses. Valid and reliable tools are needed to provide direct, cost-effective methods to assess flavonoid intake in individuals and populations [21].

Existing dietary assessment instruments may be inadequate to evaluate habitual flavonoid intake. For example, general food frequency questionnaires (FFQ) frequently group nutritionally similar foods together to reduce the number of food items (e.g., green and red grapes or berries). However, these individual food items often have distinctly different flavonoid profiles [22]. Furthermore, in population-based studies, the use of FFQs to assess usual dietary intake can be lengthy as they typically comprising 100-200 items. The time burden of such instruments often causes participants to experience frustration and fatigue, which can impede their ability to complete them. [23, 24]. More user-friendly instruments may enhance participant compliance and the accuracy of dietary data collection.

For flavonoids, an existing flavonoid-specific food frequency questionnaire (Kent & Charlton Flavonoid FFQ) [25], appears suitable for categorising individuals based on flavonoid consumption levels. However, it systematically overestimates habitual flavonoid intake against the 4-day food record, likely attributable to its extensive nature, including 96 food items. This extensive list potentially heightens reporter bias, increases participant burden, and elevates the costs associated with data collection. Moreover, the validation of this tool relied on a 4-day food record, which may not be sufficient to minimise the intra- and inter-individual variability in flavonoid intake [16] as it has been estimated that six days of weighed food record (WFR) data are needed to accurately estimate total flavonoid intake, and 6 to 10 days are required for flavonoid subclasses

There is a need to refine and further validate dietary assessment tools to assess flavonoids to ensure they are practical, valid, and feasible for accurately measuring flavonoid intake.

This study developed a shortened version (FLAV-Q) of the published 96-item Kent & Charlton Flavonoid FFQ [25] and aimed to assess its validity for measuring total flavonoid intake and a range of subclasses of flavonoids against repeated 24-h recalls in a sample of Australian adults. This study also aimed to assess the reproducibility of this tool at four timepoints over one year.

## Method and materials

### FLAV-Q development

FLAV-Q is based on an existing 96-item Kent and Charlton FFQ [25] that was specifically designed to assess the usual intake of dietary flavonoids in individuals over a period of 12 months and was originally structured using the framework of the National Health and Nutrition Examination Survey (NHANES) FFQ [26], incorporating all methodological considerations and points addressed by Cade et al. during its development and production [27, 28]. The methodology for development of this Kent and Charlton FFQ is reported in more details (see [25]). To reduce the number of food items on the original FFQ, Pearson correlations were conducted for the food items in relation to total flavonoid intake and flavonoid subclasses. Foods with positive (r > 0.10) correlation coefficients were selected for entry into regression models. Linear regression models, using the stepwise method of inclusion of variables in the forward direction, identified the food items, based on their flavonoid and flavonoid subclass composition, that did not contribute to the explanation of the total flavonoid intake and excluded these factors from the models. The remaining food items formed the basis of a new, shorter self-administered FFQ, which collected information about habitual intake of flavonoid-rich foods. Furthermore, in the initial analysis, only food items that contributed more than 1% to overall flavonoid intake were included in FLAV-Q. The new shortened Flavonoid Food Frequency Questionnaire (FLAV-Q) consists of 23 food items, divided into 3 categories, to assess an individual's habitual consumption of flavonoid-rich foods over the past 12 months. Details about the design of this tool are provided in supplementary file.

Table 1 [supplementary file] presents the correlations between all food items included in the original Kent and Charlton FFQ and total flavonoid intake, as well as each flavonoid subclass. These findings highlight the disproportionate contribution of certain foods (e.g., strawberries) to overall flavonoid estimates, thereby justifying the emphasis placed on the remaining 23 items. To ensure sufficient power for these analyses, an independent statistician (MB) calculated the required sample size based on a previous validation study in older Australians, which assessed the validity of a FFQ against multiple 24-hour recalls [29]. This calculation determined that a minimum of 42 participants would be required to detect a correlation coefficient of 0.377. Our study met this requirement, confirming that the sample size was adequate for the intended analyses

**Table 1:** Summary statistics of subject characteristics (n = 80)

Sex	Male (%) Female (%) Other (%)	25 (31.3%) 54 (67.5%) 1 (1.3%)
Age (year)	Mean ± SD	36.3 ± 11.71
Weight (kg)	Mean ± SD	68.56 ± 15.88
Height (m)	Mean ± SD	1.69 ± 0.10
BMI (kg/m^2^) Underweight Healthy Overweight Obese	Mean ± SD % % % %	23.82 ± 4.24 8.8 56.3 28.7 6.3
Supplement intake	Do not take supplements Take supplements	44 (55%) 36 (45%)
Alcohol intake (%)	Do not drink alcohol Rarely Monthly Weekly Daily	18 (22.5%) 26 (32.5%) 14 (17.5%) 19 (23.8) 3 (3.8%)
Smoking status (%)	Never smoker Previously a smoker Current smoker	83.8% 11.13% 5.0%
Employment status (%)	Employed Self-employed Unemployed Retired Homemaker/Family carer Student Other	65.0% 6.3% 3.8% 1.3% 1.3% 21.3% 1.3%
Education status (%)	Secondary school Tertiary education	5% 95%

### Study participants

This study was approved by the Western Sydney University Human Research Ethics Committee under the approval number of H14701. Inclusion criteria consisted of Australians aged 18y+; without dietary restrictions on the type and quantity of fruits and vegetables in their diet; those with Internet and email access; and who were able to provide informed consent. Exclusion criteria included pregnancy and/or breastfeeding as this may have changed dietary patterns and nutrient requirements across the study duration.

### Study design and data collection

All key recommendations for executing a high-quality validation of a dietary assessment tool [30] have been considered in the development of the current validation study, including the use of an adequately powered sample size of 50 to 100 participants that has been suggested as appropriate for relative validation studies of new dietary assessment techniques such as FFQs [27, 31], trained collection of reference dietary information, and accounting for seasonal variation in dietary intake.

Data collection occurred between March 2022 and July 2023.

Participants were recruited using convenience sampling and online through University’s official account on Facebook. provided consent by signing an agreement through the online survey and agreed to be contacted via email at four time points throughout the year, with links to online dietary surveys. All data was collected using a secure web-based application for building and managing online surveys and databases (REDCap) [32].

Using the participants’ email addresses, the first surveys were sent directly to them via REDCap, while the subsequent surveys at timepoints 2, 3 and 4 were sent automatically every three months following the first date of entry. This allowed the FLAV-Q to be tested for reproducibility at multiple timepoints over a longer period. The survey comprised the FLAV-Q, demographic, socioeconomic and self-reported anthropometric data including body mass index (BMI), age and dietary supplement use at timepoint 1. At the following timepoints FLAV-Q was completed in addition to a series of questions that characterise personas related to eating attitudes known as the “Living and Eating for Health Segments (LEHS) [33]. These questions offer a chance to explore and better understand the behaviours associated with healthy living and food choices. The details are provided in the supplementary file. At each timepoint participants were instructed by a nutritionist (EL) to self-complete two consecutive online 24-hour diet recalls using the Intake24 web-based programme.

The relative reference method for the validation study was repeated 24-h recalls conducted using Intake24 (Supplementary file). Intake24 is an open-source, self-completed computerised dietary recall system based on the multiple-pass 24-hour recall method [34] The online system offers similar data quality to interviewer-led recalls at a significantly lower cost [35] and has been validated against doubly-labelled water in a study by University of Cambridge [36]. Intake24 has been adapted for use in Australia [37] and provided accurate intake distributions in a feeding study compared to other technology-assisted methods [38].

### Calculation of flavonoid intakes

Flavonoid and flavonoid subclasses’ content were assigned for each food and beverage item included in the FLAV-Q, based on the most similar and appropriate food/beverage available in the reference PhenolExplorer [39] database. In the case of missing food items in this database, for example, mandarin and blackcurrant juice, flavonoid contents of the selected food items were identified from the USDA database [40]. Total flavonoid content and its subclasses were identified for a 100 g serve of each food item. A frequency factor, derived from the NHANES FFQ codes [26], was applied to convert pre-quantified portion sizes of foods (defined by NHANES) into daily intake values by multiplying the portion sizes by the corresponding frequency factor. For instance: 4 or more times per day = 4.5, 2–3 per day = 2.5, 1 per day = 1, 5–6 per week = 0.8, 3–4 per week = 0.5, 1–2 per week = 0.2, 2–3 per month = 0.09, and Never = 0.

Dietary data and nutrient values were exported from Intake24 into a Microsoft Excel file using Microsoft Excel for Microsoft 356 Version 2210 [41]. The data was then screened for missing food items. Daily entries with fewer than 10 food items or completed in under 2 minutes were excluded. Additionally, daily energy intakes exceeding 4000 kcal (16,738 kJ) or below 400 kcal (1,674 kJ) were excluded from the analysis. If participants reported unusual portion sizes or required clarification, they were contacted via email. Since Intake 24 did not provide data on flavonoid content, each food item (AUSNUT 2011‑13 [42]) from the 24-hour dietary recalls was manually assigned a value for total flavonoid and each subclass, based on the PhenolExplorer and USDA databases. These values were then used to calculate each participant's daily flavonoid intake.

### Statistical analysis

SPSS version 25.0 (IBM Corporation, Somers, NY, USA) was utilised for data analysis. The Kolmogorov-Smirnov and Shapiro-Wilk tests applied to total flavonoid (and flavonoid subclass) data revealed departures from a normal distribution, necessitating the use of non-parametric tests to assess validity and reproducibility. Participants who completed at least four of eight days of 24-h dietary recalls were considered eligible for validating the FLAV-Q. To account for within-individual variability in measurements of average flavonoid intakes (and flavonoid subclasses) in the 24-h dietary recalls, the Multiple Source Method (MSM) [43] was used. This online program converts actual nutrient intake to habitual intake by combining repeated 24-h dietary recalls data, making it comparable with frequency information from a long-term instrument such FLAV-Q measurements. Choosing four or more days helped to ensure the accuracy of nutrient consumption estimates, while maximising sample size. Thus, deattenuated correlations represent values corrected for within-individual variance.

First, the primary dietary sources of flavonoids and their respective subclasses were identified by determining the percentage contribution of each food item to the total flavonoid intake and to the intake of each flavonoid subclass. This was calculated for both the FLAV-Q data and the 24-h dietary recall four-day average. For each participant, the mean (SD), median, and range for total flavonoid intake and for each flavonoid subclass, was calculated for FLAV-Q data at different time points, as well as for the 24-hour dietary recalls.

Validity was tested for FLAV-Q1 versus MSM 24-h dietary recall (habitual intake) [44]; Repeatability for FLAV-Q1 was tested against all other (three) timepoints. In all comparisons, significance was considered at p < 0.05.

- The mean percentage difference was calculated by dividing the mean difference between FLAV-Q1 and the 24-h dietary recall by the mean of the 24-h dietary recall. This same method was used to find the mean percentage differences for FLAV-Q [1–4].
- A Wilcoxon signed-rank sum test was used to compare flavonoid and flavonoid subclass intakes obtained from the two methods (FLAV-Q1 versus habitual intake (multiple 24-h recalls)), as well as FLAV-Q [1–4].
- The Spearman’s correlation test was applied to assess the strength of association for flavonoid and its subclasses between the two dietary methods for validity and reproducibility at four timepoints. Correlation coefficients less than 0.20 were considered poor, those between 0.20 and 0.49 acceptable, and those 0.50 or higher were considered good outcomes [44].
- Bland–Altman plots were used to evaluate the level of agreement between the two methods. For these plots, the difference between FLAV-Q1 and habitual intake (multiple 24-h recalls) (FLAV-Q1 – habitual intake) was plotted against the mean of FLAV-Q1 and habitual intake ((FLAV-Q1 + habitual intake / 2). Bland–Altman graphs were also constructed to examine the differences between FLAV-Q [1–4]. Limits of agreement (LOA; the mean difference ± 1.96SD) for the difference between the two measures were calculated to evaluate their acceptability. A regression line was fitted to detect proportional differences and to indicate the direction and magnitude of bias. The Bland–Altman index was calculated as the percentage of paired observations with differences exceeding the 95% limits of agreement which when below 5% was interpreted as good, as suggested by previous research [45–47]. While log transformation is often recommended for skewed data in Bland–Altman analyses, the original scale of measurement (mg/day) was retained to facilitate the interpretation of absolute bias and limits of agreement in flavonoid intake.
- Cohen's kappa (κ) test was used to determine the ability of the FLAV-Q to rank individuals according to quartiles of intake (FLAV-Q1 versus. habitual intake (multiple 24-h recalls) and FLAV-Q1 versus. FLAV-Q2,3,4) for total flavonoid intake and flavonoid subclasses [48, 49]. Values of 0.00 – 0.20 were considered slight agreement, 0.21 – 0.40 fair agreement, 0.41 – 0.60 moderate agreement, 0.61 – 0.80 substantial agreement, and 0.81 – 1.00 almost perfect agreement.

## Results

**Fig. 1:**
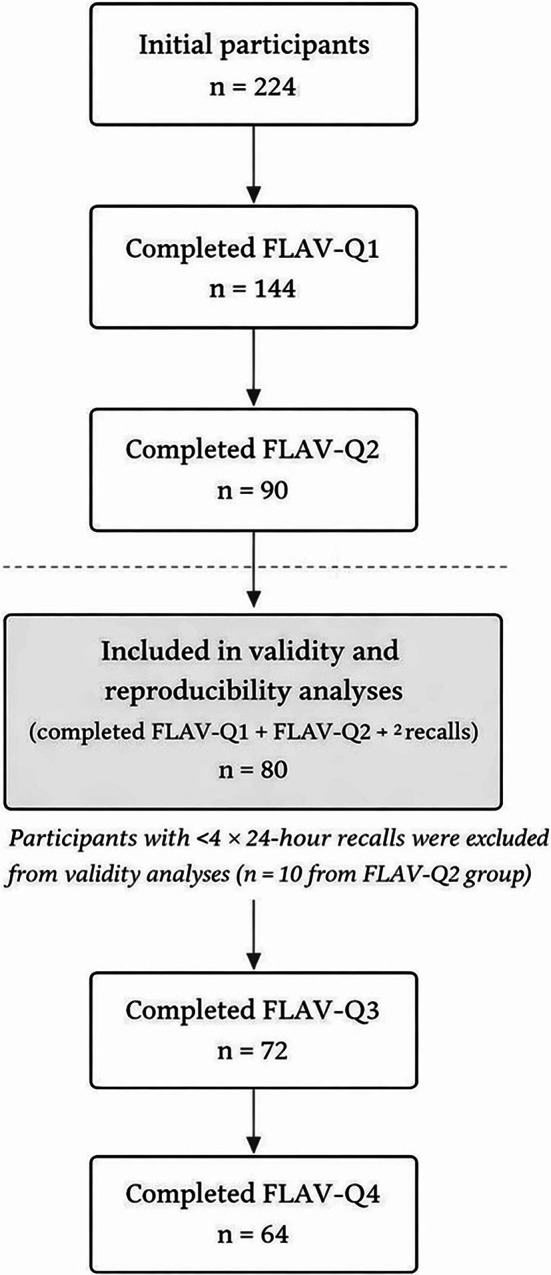
Participant flowchart diagram. Number of participants completing each timepoints (FLAV-Q1, FLAV-Q2, FLAV-Q3, FLAV-Q4) and their respective 24-h dietary recall

Figure 1 demonstrates participant inclusion and follow- up. Of the 224 eligible participants who attempted the survey, 144 completed FLAV-Q1 and 90 completed FLAV-Q2. A total of 80 participants who completed both FLAV-Q1 and FLAV-Q2 and had ≥4 24-h dietary recalls were included in the validity and reproducibility analyses. Subsequent completions of FLAV-Q3 and FLAV-Q4 reflect follow-up participation only.

The demographic characteristics of the participants are detailed in Table 1. The majority of participants were women (67.5%) with a mean age of 36.3 years (SD = 11.71) and age range of 30-85 years old and a mean BMI of 23.82 kg/m^2^ (SD = 4.24) with almost half of the participants (56.3%) being in healthy range. Over half (55%) of participants reported zero or infrequent alcohol consumption, while most were non-smokers (83.8%). Additionally, 65% of the participants were employed, and 95% had attained tertiary education.

## Validation (FLAV-Q1 versus habitual intake (multiple 24-h recalls))

Data from FLAV-Q1 and the habitual intake are presented in Table 2. There was substantial variability in dietary flavonoid intake and the intake of flavonoid subclasses between methods, as indicated by the wide range of intakes observed. On average, the flavonoid intake reported in FLAV-Q1 was statistically higher compared to habitual intake, with a mean percentage difference of 89.05% between the two methods. The mean percentage difference for the subclasses: anthocyanins, flavan-3-ols, flavanones, flavones and flavonols was reported to be approximately higher by 232.28%, 56.01%, 34.26%, 57.03% and 251.62% respectively in FLAV-Q1 compared to habitual intake.

**Table 2:** Description of mean flavonoids intake and its subclasses (mg/day) according to the FLAV-Q1 and habitual intake (n = 80)

	FLAV-Q1	Habitual intake*	Mean difference (FLAV-Q1—habitual intake)	Mean percentage difference
*Total flavonoid (mg/day)*				
Mean ± SD	443.24 ± 373.35	234.44 ± 214.14)	208.81	89.05%
Median	293.77	142.00		
Minimum	22.80	8.16		
Maximum	1600.79	751.61		
% contribution	–	–		
*Anthocyanins (mg/day)*				
Mean ± SD	72.67 ± 129.18	21.87 ± 27.60	50.80	232.28%
Median	34.84	11.19		
Minimum	0.00	0.19		
Maximum	913.66	152.03		
% contribution	16.40%	9%		
*Flavan-3-ols (mg/day)*				
Mean ± SD	262.39 ± 286.00	168.18 ± 185.05	94.21	56.01%
Median	150.45	86.32		
Minimum	3.67	4.02		
Maximum	1286.37	667.31		
% contribution	59.14%	71.74%		
*Flavanones (mg/day)*				
Mean ± SD	25.81 ± 36.83	39.61 ± 92.65		
Median	10.91	7.45	-13.57	-34.26%
Minimum	0.00	0.07		
Maximum	197.82	639.24		
% contribution	5.82%	16.90%		
*Flavones (mg/day)*				
Mean ± SD	4.13 ± 5.91	2.63 ± 5.15	1.50	57.03%
Median	2.56	1.05		
Minimum	0.06	0.05		
Maximum	34.91	31.27		
% contribution	0.93%	1.12%		
*Flavonols (mg/day)*				
Mean ± SD	77.44 ± 65.52	22.03 ± 19.71	55.41	251.62%
Median	60.07	14.60		
Minimum	8.39	1.33		
Maximum	446.77	96.13		
% contribution	17.47%	9.40%		

^*^Habitual intake was assessed using the Multiple Source Method (MSM), % Contribution = (FLAV-Q1 intake – Habitual intake) / Habitual intake × 100

The primary sources of flavonoids are shown in Table 3. Black tea was the top source for total flavonoid intake consumed both habitually (57.70% total) and according to the FLAV-Q1 (44.41%). Blueberries were reported as the main source of anthocyanin intake for both methods (habitual intake: 36.82% versus FLAV-Q1: 52.88%); black tea was the highest source of flavan-3-ols (habitual intake: 76.49% versus FLAV-Q1: 66.99%) and flavonols (habitual intake: 43.27% versus FLAV-Q1: 30.41%); and orange juice was the leading source of flavones for habitual intake (38.78%) whereas parsley was the top source of flavones according to FLAV-Q1 (45.26%). Additionally, the highest food source for flavanone was determined to be orange juice for habitual intake (61.40%) and orange for FLAV-Q1 (50.14%).

**Table 3:** Top 10 foods contributing to total flavonoid and flavonoid subclasses intake (mg/day %) ranked in order of contribution to total consumption according to habitual intake and FLAV-Q1

		Habitual intake*		FLAV-Q1	
Total flavonoid		Food	%	Food	%
	1	Black tea	57.70	Black Tea	44.41
	2	Green tea	6.57	Blueberry	11.21
	3	Orange juice	4.81	Green Tea	10.05
	4	Blueberry	3.87	Beans	6.37
	5	Other herbal tea	3.72	Cherry	4.43
	6	Red wine	3.42	Red apple	3.22
	7	Chocolate	3.26	Red wine	3.11
	8	Red apple	1.65	Orange	2.91
	9	Oranges	1.51	Red grapes	2.50
	10	Spinach	1.17	Orange Juice	2.46
Anthocyanins	1	Blueberry	36.82	Blueberry	52.88
	2	Strawberry	12.26	Cherries	24.91
	3	Red wine	11.41	Red grapes	12.39
	4	Red cabbage	9.26	Red wine	5.15
	5	Black beans	5.14	Blackcurrant juice	2.47
	6	Red grapes	3.92	Red onion	1.44
	7	Mixed berries	3.88	Red apple	0.49
	8	Cherry juice	3.81		
	9	Cranberry juice	2.86		
	10	Cherry	2.55		
Flavan-3-ols	1	Black tea	76.49	Black tea	66.99
	2	Green tea	8.74	Green tea	15.92
	3	Chocolate	4.19	Beans	8.60
	4	Other herbal tea	3.03	Red apple	3.53
	5	Red wine	2.69	Red wine	3.01
	6	Red apple	2.04	Red grape	0.67
	7	Kiwi	1.20	Cherry	0.61
	8	Prune juice	0.25	Pear	0.27
	9	Plum	0.24	Green grape	0.26
	10	Banana	0.22	Blueberry	0.12
Flavones	1	Orange juice	38.78	Parsley	45.26
	2	Parsley	27.44	Celery	19.02
	3	Pumpkin	8.49	Orange Juice	16.34
	4	Celery	5.00	Spinach	13.95
	5	Melon	2.87	Beans	2.58
	6	Spinach	2.09	Red apple	1.58
	7	Bok choy	2.06	Red onion	1.30
	8	Lemon	1.48		
	9	Red apple	1.28		
	10	Capsicum	0.97		
Flavanones	1	Orange juice	61.40	Orange	50.14
	2	Oranges	20.57	Orange Juice	39.88
	3	Mandarin	6.71	Mandarin	10.31
	4	Grapefruit juice	2.47	Red wine	0.56
	5	Tomatoes	2.0		
	6	Red wine	0.49		
	7	Tropical juice	0.37		
	8	Beer	0.08		
	9	White wine	0.06		
	10	Almonds	0.03		
Flavonols	1	Black tea	43.27	Black tea	30.41
	2	Spinach	12.78	Blueberry	14.37
	3	Blueberry	9.59	Broccoli	12.41
	4	Asparagus	6.53	Spinach	9.07
	5	Red wine	3.16	Brown onion	7.85
	6	Green tea	2.85	Beans	7.47
	7	Chocolate	2.59	Kale	5.14
	8	Tomatoes	1.99	Green tea	4.00
	9	Kale	1.90	Red apple	3.37
	10	Walnut	1.67	Red onion	2.92

^*^*Habitual intake was assessed using the Multiple Source Method (MSM)

Generally, the main food sources for flavonoid and its subclasses are similar between the two methods, with FLAV-Q1 capturing most of the key sources included in habitual dietary intake. There were however some food sources identified in the habitual intake data that were missing from FLAV-Q such as strawberries (provided 12.26% of anthocyanins), other herbal teas (total flavonoid: 3.72%), chocolate (flavan-3-ol: 4.19%) and pumpkin (flavones: 8.49%).

The Wilcoxon signed-rank sum test (Table 4) showed significant differences in total flavonoid, total anthocyanins, total flavan-3-ols, total flavones, total flavonol intakes as measured by the FLAV-Q1 and habitual intake (multiple 24-h recalls). A strong, significant and positive correlation was found between FLAV-Q1 and habitual intake for total flavonoid intake, flavan-3-ols and flavonols. Correlations were moderate and significant for flavanones, anthocyanins and flavones. Cohen’s kappa (κ) test results showed moderate agreement between the two methods for total flavonoids intake and the subclasses of flavan-3-ols and flavonols, and fair agreement for flavanones intake. For anthocyanins and flavones, the agreement was only slight and was non-significant.

**Table 4:** Comparison of the total flavonoid intake and intake of flavonoid subclasses (mg/day) for FLAV-Q-1 versus habitual intake (multiple 24-h recalls)

	Wilcoxon signed-rank sum test sig z-value(*p*-value)	Spearman’s correlation coefficient sig. (*p*-value)	Cohen’s Kappa (κ) sig (*p*-value)
Total flavonoids	6.22 (0.001)	0.66 (< 0.001)	0.45 (< 0.001)
Anthocyanins	5.70 (< 0.001)	0.38 (< 0.001)	0.15 (0.18)
Flavan-3-ols	4.02 (< 0.001)	0.72 (< 0.001)	0.53 (< 0.001)
Flavanones	0.02 (0.99)	0.49 (< 0.001)	0.30 (0.007)
Flavones	4.06 (< 0.001)	0.34 (< 0.001)	0.20 (0.07)
Flavonols	7.76 (< 0.001)	0.55 (< 0.001)	0.4 (< 0.001)

**Fig. 2:**
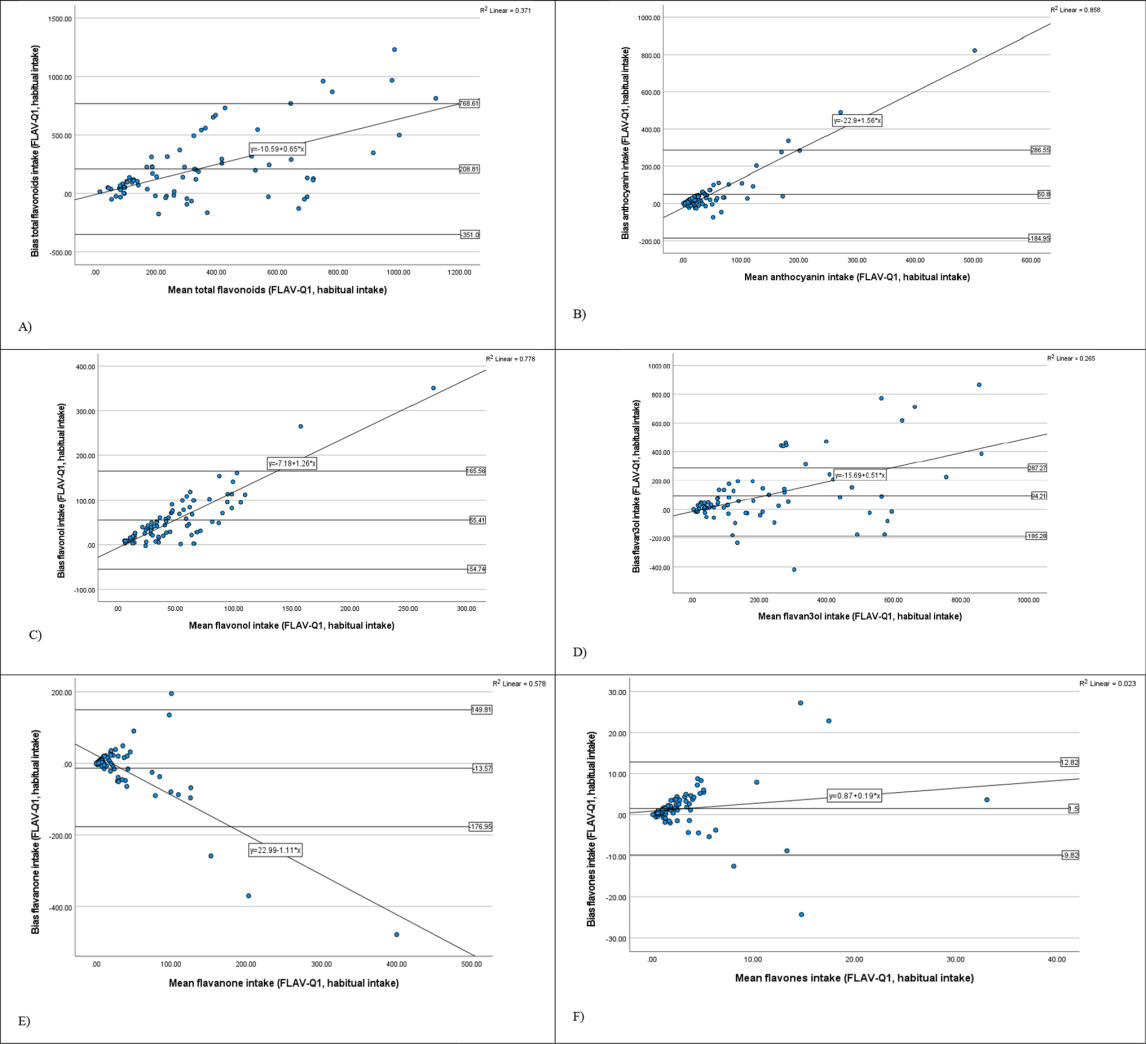
Bland–Altman plots (difference in intake (mg/day) (FLAV-Q1—habitual intake) against the mean intake of flavonoids and subclasses (mg/day) [FLAV-Q1 + habitual intake/2]) showing the relative validity of the FLAV-Q1 versus the habitual intake for total (**A**) flavonoids, (**B**) anthocyanin, (**C**) flavonol, (**D**) flavan-3-ol, (**E**) flavanone and (**F**) flavone intake

The Bland–Altman plots illustrating bias are shown in Fig. 2. According to the plot, the data is mostly clustered around the mean bias with wide LOA, further suggesting large bias for total flavonoid intake (mean bias =  + 208.81 mg/day, 95% CI = -351, 768, Bland–Altman index: 7.5%) and medium bias for anthocyanins (mean bias =  + 50.8 mg/day, 95% CI = -184.95, 286.55, Bland–Altman index: 3.8%), flavonol (mean bias =  + 55.41 mg/day, 95% CI = 54.74, 165.68, Bland–Altman index: 2.5%) and flavan-3-ol (mean bias =  + 84.21 mg/day, 95% CI = -185.28, 287.27, Bland–Altman index: 13.8%). The positive slopes suggest increased overestimation by FLAV-Q1 at higher habitual intakes (multiple 24-h recalls). Flavanone intake (mean bias = -13.57 mg/day, 95% CI = -176.95, 149.81, Bland–Altman index: 1.3%) had a smaller bias with a negative slope, indicating the difference between FLAV-Q1 and habitual intake becomes more negative with increasing intake. The bias for flavones was small with narrow limits of agreement (mean bias value =  + 1.5 mg/day, 95% CI = -9.82,12.82, Bland–Altman index: 2.5%).

Furthermore, the linear regression identified in the Bland-Altman plots indicates moderate to strong positive correlation for flavon3ol (R^2^ = 0.27), total flavonoid (R^2^ = 0.37), flavanone (R^2^ = 0.58), flavonol (R^2^ = 0.78) and anthocyanin (R^2^ = 0.86), suggesting that as the intake increases, bias becomes larger in a moderate to strong predictable way, for total flavonoids and their subclasses. Weaker linear regressions for flavones (R^2^ = 0.02) suggest no systematic bias for this subclass, however, the variation in bias increases as the mean flavones increases. There is increasing variability in most plots as the mean intakes increase and the slopes are influenced by high outlying intakes.

### Reproducibility (FLAV-Q1 versus FLAV-Q2, FLAV-Q3 and FLAV-Q4)

Table 5 demonstrates the variability in dietary flavonoid intake and flavonoid subclasses between timepoints. The mean percentage difference between total flavonoid intake at timepoint 1 (FLAV-Q1) compared to the three subsequent timepoints (FLAV-Q2, FLAV-Q3, and FLAV-Q4) was higher by 22%, 36.9%, and 34%, respectively. Moreover, FLAV-Q1 reported higher intake values for total anthocyanin, flavan-3-ols, flavones and flavonols intake compared to the three other timepoints. Flavanone intake was similar between FLAV-Q1 and FLAV-Q2, however, it was higher at FLAV-Q1 compared to FLAV-Q3 and FLAV-Q4 by 8.5% and 6.3%, respectively.

**Table 5:** Description of mean flavonoid intake (mg/day) according to the FLAV-Q1 versus FLAV-Q2, FLAV-Q3 and FLAV-Q4

	FLAV-Q1 N = 80	FLAV-Q2 N = 80	Mean difference (FLAV-Q1—FLAV-Q2)	Mean percentage (%) difference	FLAV-Q3 N = 72	Mean difference (FLAV-Q1—FLAV-Q3)	Mean percentage (%) difference	FLAV-Q4 N = 64	Mean difference (FLAV-Q1—FLAV-Q4)	Mean percentage (%) difference
*Total flavonoid(mg/day)*										
Mean ± SD	443.24 ± 373.35	363.48 ± 280.93	79.76	21.94%	323.72 ± 285.31	119.52	36.92%	353.16 ± 294.47	120.08	34.00%
Median	293.77									
Minimum	22.80	268.14			212.74			249.04		
Maximum	1600.79	36.50			28.87			43.66		
% contribution	–	1110.14			1261.36			1202.09		
		–			–			–		
*Total Anthocyanins*										
(mg/day)						27.06	59.33%	49.29 ± 84.00 25.69 3.51	23.38	47.43%
Mean ± SD	72.67 ± 129.18	52.25 ± 72.68	20.42	39.08%	45.61 ± 57.84					
Median	34.84	23.44								
Minimum	0.00	0.33			22.93			516.78		
Maximum	913.66	388.81			1.84			13.96%		
%	16.40%	14.37%			250.88					
contribution					14.09%					
*Total Flavan-3-ols*										
(mg/day) Mean ± SD	262.39 ± 286.00	220.94 ± 232.62	41.45	18.76%	195.45 ± 231.79	66.94	34.25%	220.40 ± 243.16 126.01	41.99	19.05%
Median	150.45									
Minimum	3.67	114.88			94.70			1.74		
Maximum	1286.37	1.19			6.03			874.75		
% contribution	59.14%	911.00			873.53			62.41%		
		60.78%			60.38%					
*Total Flavanones*										
Mean ± SD	25.81 ± 36.83	26.07 ± 31.14	-0.26	1%	23.80 ± 31.51	2.01	8.45%	24.28 ± 34.03	1.53	6.30%
Median	10.91	14.20						11.91		
Minimum	0.00	0.00			11.84			0.00		
Maximum	197.82	183.87			0.00			175.94		
% contribution	5.82%	7.17%			153.31			6.88%		
					7.35%					
*Total Flavones*										
(mg/day)				18%	2.98 ± 3.17	1.15	38.59%	3.94 ± 4.89 2.40 0.06	0.19	4.82%
Mean ± SD	4.13 ± 5.91	3.50 ± 4.13	0.63							
Median	2.56	2.20			1.72					
Minimum	0.06	0.06			0.02			27.83		
Maximum	34.91	26.46			14.13			1.12%		
% contribution	0.93%	0.96%			0.92%					
*Total Flavonols*										
Mean ± SD	77.44 ± 65.52	59.19 ± 37.53	18.25	30.83%	55.18 ± 39.88	22.26	40.34%	55.18 ± 36.73	22.26	40.34%
Median	60.07	52.81						45.5201		
Minimum	8.39	7.02			43.87			5.31		
Maximum	446.77	173.61			6.05			181.11		
% contribution	17.47%	16.28%			207.68			15.62%		
					17.05%					

The significant differences in total flavonoid intake between FLAV-Q1 and subsequent time points are shown in Table 6. According to the Wilcoxon signed-rank test, total flavones intake was significantly higher in FLAV-Q1 compared to FLAV-Q3 as was flavonol intake.

**Table 6:** Comparison of the total flavonoid intake and intake of flavonoid subclasses (mg/day) for FLAV-Q-1 versus FLAV-Q2, FLAV-Q3 and FLAV-Q4 (reproducibility)

		Wilcoxon signed-rank sum test sig z-value (*p*-value)	Spearman’s correlation coefficient (*p*-value)	Cohen’s Kappa (κ) sig (*p*-value)
FLAV-Q1 versus FLAV-Q2	Total flavonoid	2.29 (0.02)	0.82 (< 0.001)	0.70 (< 0.001)
	Anthocyanins	1.97 (0.04)	0.55 (< 0.001)	0.35 (0.002)
	Flavan-3-ols	1.54 (0.12)	0.85 (< 0.001)	0.65 (< 0.001)
	Flavanones	− 0.84 (0.40)	0.76 (< 0.001)	–
	Flavones	0.93 (0.35)	0.59 (< 0.001)	0.30 (0.007)
	Flavonols	2.87 (0.004)	0.60 (< 0.001)	0.43 (< 0.001)
FLAV-Q1 versus FLAV-Q3	Total flavonoid	3.01 (0.003)	0.68 (< 0.001)	0.47 (< 0.001)
	Anthocyanins	0.95 (0.35)	0.40 (< 0.001)	0.20 (0.07)
	Flavan-3-ols	3.25 (0.001)	0.79 (< 0.001)	0.50 (< 0.001)
	Flavanones	0.58 (0.56)	0.63 (< 0.001)	0.00 (1.00)
	Flavones	2.33 (0.02)	0.59 (< 0.001)	0.33 (0.005)
	Flavonols	2.58 (0.01)	0.49 (< 0.001)	0.25 (0.03)
FLAV-Q1 versus FLAV-Q4	Total flavonoid	2.54 (0.01)	0.63 (< 0.001)	0.47 (< 0.001)
	Anthocyanins	2.0 (0.04)	0.45 (< 0.001)	0.20 (0.07)
	Flavan-3-ols	2.10 (0.04)	0.75 (< 0.001)	0.63 (< 0.001)
	Flavanones	− 0.96 (0.34)	0.69 (< 0.001)	–
	Flavones	0.21 (0.83)	0.58 (< 0.001)	0.47 (< 0.001)
	Flavonols	2.220 (0.02)	0.41 (< 0.001)	0.34 (0.006)

Cohen's kappa (κ) test indicates substantial agreement between FLAV-Q1 and FLAV-Q2 for total flavonoid and flavan-3-ol, moderate agreement for flavonol and fair agreement for anthocyanins and flavones. Moderate to slight agreement was observed between FLAV-Q1 and FLAV-Q3 for total flavonoid intake, flavan-3-ols, flavones and flavonols. For FLAV-Q1 and FLAV-Q4, fair agreement was reported for flavonols, moderate agreement for total flavonoids and flavones, and substantial agreement for flavan-3-ols

The strength of association between FLAV-Q1 and the subsequent timepoints is shown in Table 6. These results suggest a good, significant association in total flavonoid intake between FLAV-Q1 and each of the subsequent timepoints. For anthocyanin intake, Spearman’s correlations indicated good and significant associations for FLAV- Q1 versus FLAV-Q2, and moderate associations for FLAV-Q1 versus FLAV-Q3 and for FLAV-Q1 versus FLAV-Q4. Total flavan-3-ols also showed significant and strong correlations between FLAV-Q1 and each of the other timepoints. The association for total flavanones was significant and strong between FLAV-Q1 and the other 3 timepoints. Total flavones showed statistically significant association between FLAV-Q1 versus FLAV-Q 2, 3 and 4. The association for total flavonols was strong and significant between FLAV-Q1 and FLAV-Q2, acceptable for FLAV-Q1 and FLAV-Q3 and for FLAV-Q1 and FLAV-Q4.

The Bland-Altman plot, illustrating the bias between mean value for FLAV-Q1 and FLAV-Q2 for total flavonoids and the subclasses, is presented in supplementary file Figure 3 and Figure 4. Bland-Altman plots for the remaining time points are provided in supplementary file Figure 4.

## Discussion

This study is the first to develop, validate and test the reproducibility of a short flavonoid-specific FFQ (FLAV-Q) comprising 23 food items in a sample of Australian adults. The FLAV-Q tool has demonstrated moderate to low validity against habitual intake (repeated 24-h dietary recalls) and reproducibility over 12 months for assessing the intake of total flavonoid and its subclasses.

The FLAV-Q estimated the average flavonoid intake to be almost double that (443 mg/day) of habitual intake (234 mg/day), but comparatively lower than the average intake reported in two recent Australian studies [15, 25]. Both these studies were conducted in older samples (39-65 years old) than the current sample (mean age (SD) = 36.3 (11.7) y) with one using FFQ [15] and the other a four day food record questionnaire [25]. The average flavonoid intake from the current study are more similar to the studies measuring the average intake of flavonoids in younger populations and using repeated 24-h dietary recalls as a method of measurement [50–52]. Each flavonoid subclass’s percentage of contribution reported in both methods (FLAV-Q and habitual intake) is in line with previous evidence [25]. Flavan-3-ol intake has been consistently reported as the highest (59.14%) out of total flavonoids, indicating black tea as the major dietary flavonoid source consumed in Australia [50].

Both FLAV-Q and habitual intake (multiple 24-h recalls) reveal similar food sources as major contributors to daily flavonoid intake. The most commonly consumed flavonoid-rich foods include black tea, green tea and blueberries [50]. Parsley was identified as a major source of flavones according to the FLAV-Q, as identified in another study [51], however, given this food is sporadically consumed in small doses, it was not detected as a major flavone food source according to habitual intake.

Although according to Spearman’s correlation test, FLAV-Q is highly correlated to habitual intake (multiple 24-h recalls) for total flavonoid intake, there is moderate agreement between the two methods with systematic overestimation reported by FLAV-Q . This discrepancy aligns with findings from previous validation evidence of FFQs against repeated 24-h recalls [50]. Such differences may be attributed to recall biases (which can lead to overestimation or under estimation when completing a 24-h dietary recall or a FFQ) [53], alteration in participants’ eating patterns [33, 54], intake related biases such as error in reporting quantity measurements of foods [55] and social desirability [56–58]. Furthermore, the repeated consumption of these food items throughout the day can make it easier to overreport in FFQs [59].

Regarding flavonoid subclasses, flavan-3-ols exhibited the strongest validity with the highest correlation and agreement between the FLAV-Q1 and habitual intake according to the Spearman’s correlation coefficient and Cohen’s kappa tests. Given that moderate validity was seen for flavonols and flavanones (with flavanones being underestimated) and weak validity for anthocyanins and flavones, the FLAV-Q tool is more appropriate for assessing total flavonoid intake rather than individual subclasses. Similarly to the present study, moderate validation was reported in an Australian population that included five flavonoid subclasses and a 62-item food frequency questionnaire [60] as well as a study assessing the intake of six flavonoid subclasses in American adults [61]. Another study in China also performed a validation study for a flavonoid FFQ including 12 food categories and 147 items, which further showed strong correlation coefficients according to the Spearman’s test (r=0.100~0.640) similar to the present study [62, 63]. In contrast to our results however, higher agreements for flavone and flavonol intakes have been reported [64–66]. However, these international studies typically had larger sample sizes, and one was conducted in an adolescent population [65]. Additionally, these studies used food frequency questionnaires comprising 98 to 138 food items to capture total dietary intake rather than flavonoids alone, compared to our much shorter questionnaire of only 23 food items. Moreover, similar studies employing a flavonoid-specific FFQ often utilized weighed dietary records instead of repeated 24-h dietary recalls as the standard method for FFQ validation [25, 61], resulting in stronger correlations reported between the two methods. However, according to a systematic review, the most commonly used reference method for validation of flavonoid-focused FFQs is multiple 24-h dietary recalls, due to their ability to accurately capture daily consumption of a varied diet and their relatively straightforward administration and analysis compared to other dietary methods [67].

The Bland-Altman plots in the present study revealed a systematic bias, showing that differences between the two methods increased as intake increased. This bias does not compromise the ability of FLAV-Q to rank foods contributing to flavonoid intake and reflects the semiquantitative nature of FFQs [68, 69]. The presence of outliers in all the plots suggest underlying issues, which could be related to inaccuracies in participants’ dietary recall or misreporting stemming from potential social desirability bias, or misunderstandings of the questionnaire. Furthermore, the consistent presence of outliers across all subclasses may indicate an unusual dietary pattern among some participants, characterized by exceptionally high or unique flavonoid intake due to their dietary habits. As intake levels reported in the FLAV-Q increase, there is less agreement between the two methods, indicating that the FLAV-Q may not be a valid tool for assessing higher intakes of flavonoids. This is particularly true for the subclass anthocyanin, flavonol and flavanone. Berries and cherries are rich in anthocyanin and mostly consumed during summer and spring whereas citrus fruits rich in flavanone and flavonol are specific to winter. Therefore, these fruits have a highly variable pattern of consumption throughout the year. Additionally, flavone consumption is underreported by habitual intake, likely influenced by its sporadic consumption and/or seasonality.

In terms of reproducibility, the FLAV-Q tool showed moderate reproducibility for total flavonoid. However, while there was better agreement between FLAV-Q1 and FLAV-Q2 in estimating total flavonoid intake and subclasses according to the Bland-Altman's plot and correlation coefficients there is lower agreement between the repeated FLAV-Qs (timepoint 3 and 4 conducted 6 and 9 months later, respectively). Flavanone intakes had the greatest agreement (moderate to high) and were the most consistent throughout the duration of the study. Several other studies on FFQs have reported similar findings [70, 71]. This could be partly explained by the participants’ attention to complete the survey. Another possible reason is that the FLAV-Qs were completed by participants 3-4 months apart to consider the seasonal variation in the diet. As mentioned above for validity, flavonoid food sources’ availability such as blueberries are highly seasonal. FLAV-Q at timepoint 1 was different for each participant as some commenced the study in summer while others started during winter, leading to variation in intakes at each timepoint. Despite the discrepancies in reporting major food sources of total flavonoids and their subclasses, adding certain food items to the FLAV-Q may enhance future studies. Food items that were missing from the FLAV-Q include: strawberries, red cabbage, mixed berries, and cranberry juice (rich in anthocyanins); bananas and chocolate (high in flavan-3-ols); celery, pumpkin, melon, and lemon (good sources of flavones); and asparagus, chocolate, tomatoes, and walnuts (flavonols).

The strengths of this validation study include characteristics that makes the FLAV-Q particularly useful for time-limited surveys where detailed dietary assessments are not feasible. Another strength is the study's design, which included an adequate number of repeated 24-h dietary recalls to estimate long-term dietary intake [72] and transformation of actual dietary intake into habitual intake using the MSM software which removes the effect of day-to-day variability in the average estimates of repeated 24-h dietary recalls. The food list for the FLAV-Q was constructed with consideration for seasonal variations in food intake within this population. The validation study was conducted from summer to winter, covering the period of seasonal food variation, particularly for fruits and vegetables. Finally, the sample size of the study was ideal for achieving a desirable level of validation, according to previous literature [27].

Several potential limitations should be considered. The nature of self-completing the survey questionnaire may have resulted in reporter bias due to mistakes in completing the survey without specialist supervision (34). Additionally, the performance of the FLAV-Q varied across different seasons, reflecting fluctuations in actual intake, which may account for the significant differences observed between FLAV-Q measurements at various time points. A further limitation is the absence of intraclass correlation coefficient (ICC) analysis, which was not conducted due to the substantial day-to-day and seasonal variability in dietary intake—particularly for foods consumed episodically or irregularly. Such variability can result in low ICC values that may not accurately represent the validity of the dietary assessment method. The use of the USDA and PhenolExplorer databases to determine the flavonoid content of selected foods as a reference database may be another limitation as the flavonoid content of foods in these databases may not accurately reflect that of Australian produce, as flavonoid content is heavily influenced by cultivar, growth conditions, and processing methods [73]. PhenolExplorer exhibits limitations in data availability for certain polyphenols such as proanthocyanins and does not provide information on more specific food items such as distinct cultivars of fruits or different coffee brews. This limitation arises from the insufficient number of compositional data points available in the literature, which impedes the ability to accurately compare the concentrations of various polyphenols in these specific foods. However, PhenolExplorer is a more reliable tool than USDA. Furthermore, the use of convenience sampling via an online platform may limit the generalisability of the findings, as the sample may not be representative of the broader population. Finally, no suitable biomarkers were used to validate flavonoid intake, as the study assessed multiple flavonoid subclasses for which no single biomarker exists that can comprehensively reflect their intake. The identification of reliable and subclass-specific biomarkers remains beyond the scope of the current study and represents an important direction for future research.

In conclusion, the newly developed FLAV-Q, designed to estimate flavonoid intake in Australian adults, demonstrates satisfactory performance in ranking individuals based on their total flavonoid intake and ranking food sources of flavonoids in comparison to a measure of habitual intake derived from repeated 24-h dietary recalls. However, it exhibits limitations in accurately estimating absolute flavonoid intake, particularly for specific flavonoid subclasses, as it systematically overestimates intake, particularly at high intake levels. The FFQ shows potential for estimating total flavonoid intake and subclass consumption in adults, especially in epidemiological studies where ranking is critical, further validation is necessary. Future research should focus on adjusting for overreporting to enhance the validity of the FFQs as reference methods.

## Supplementary Information

Below is the link to the electronic supplementary material.

Supplementary Material 1

## Data Availability

The datasets generated during and/or analysed during the current study are available from the corresponding author on reasonable request.
