# Supplementary material for: Validation and reproducibility of a novel flavonoid food frequency dietary assessment tool (Flav-Q) against multiple 24-h recalls across 12 months
Source: Eur J Nutr. 2026 May 26;65(4):141. doi: 10.1007/s00394-026-03997-7 (PMC13212611; doi:10.1007/s00394-026-03997-7)
Supplement: Supplementary file 1 — Supplementary Material 1 [file 394_2026_3997_MOESM1_ESM.docx]

Validation and reproducibility of a novel flavonoid food frequency dietary assessment tool (Flav-Q) against multiple 24hr- recalls across 12 months

Authors:

E. Lorzadeh 1, K. Charlton 1, T.A. McCaffrey 3, K. Weston-Green 1,2, M. Batterham4, K. Kent 1,5

Affiliations:

1. School of Medical, Indigenous and Health Sciences, Faculty of Science, Medicine and Health, University of Wollongong, NSW, 2522, Australia.

2. Molecular Horizons, Faculty of Science, Medicine and Health, University of Wollongong, NSW, 2522, Australia.

3. Department of Nutrition, Dietetics and Food, School of Clinical Sciences, Monash University, Melbourne, VIC 3168, Australia.

4. School of Mathematics and Applied Statistics, Statistical Consulting Centre, University of Wollongong, NSW, 2500

5. School of Health Sciences, Western Sydney University, Campbelltown, NSW, 2560, Australia

*Corresponding Author: Dr Katherine Kent

Postal address: School of Medical, Indigenous and Health Sciences, University of Wollongong, Northfields Ave Wollongong, NSW 2522 Australia

Email address: [katherinek@uow.edu.au](mailto:katherinek@uow.edu.au)

Supplementary file

**Table 1** Contribution and correlations of individual FFQ food items to total and subclass flavonoid intake

| Food Item | Total flavonoid | Anthocyanin | Flavan-3-ols (r)  (P value) | Flavones (r)  P-value | Flavanones (r)  P-value | Flavonols (r)  P value |
| --- | --- | --- | --- | --- | --- | --- |
| Black Tea  Correlation coefficient (r)  P value  % contribution | 0.86  (0.0)  56.97 | - | 0.86  (0.0)  75.22 | - | - | 0.71  (0.0)  29.03 |
| Green Tea  Correlation coefficient (r)  P value  % contribution | 0.43  (0.01)  6.05 | - | 0.45  (0.003)  7.42 | 0.05  (0.76)  4.22 | - | 0.39  (0.01)  3.81 |
| Coffee  Correlation coefficient (r)  P value  % contribution | -0.286  (0.07)  0.18 | - | -0.268  (0.09)  0.17 | - | - | -0.28  (0.07)  0.84 |
| Hot chocolate  Correlation coefficient (r)  P value  % contribution | -0.11  (0.51)  0.11 | - | -0.02  (0.93)  0.21 |  |  | 0.03  (0.87)  0.10 |
| Orange Juice  Correlation coefficient (r)  P value  % contribution | 0.1  (0.62)  1.0 |  |  |  | 0.37  (0.02)  17.59 | -0.02  (0.91)  0.18 |
| Apple Juice  Correlation coefficient (r)  P value  % contribution | 0.03  (0.87)  0.25 | -0.07  (0.34)  0.01 | 0.003  (0.99)  0.50 |  |  | 0.005  (0.98)  0.19 |
| Blackcurrant Juice  Correlation coefficient (r)  P value  % contribution | 0.87  (0.03)  1.27 | 0.38  (0.01)  1.17 |  |  |  | -0.04  (0.82)  0.22 |
| Lemon Juice  Correlation coefficient (r)  P value  % contribution | -0.04  (0.82)  0.07 |  |  |  | 0.08  (0.61)  0.67 | -0.08  (0.62)  0.01 |
| Beer  Correlation coefficient (r)  P value  % contribution | -0.02  (0.90)  0.11 |  | -0.06  (0.72)  0.15 |  |  | -0.01  (0.94)  0.41 |
| White wine  Correlation coefficient (r)  P value  % contribution | 0.72  (0.00)  0.37 | -0.18  (0.13)  0.24 | -0.14  (0.36)  0.37 |  | -0.1  (0.5)  3.64 | 0.11  (0.48)  0.23 |
| Red wine  Correlation coefficient (r)  P value  % contribution | 0.98  (0.004)  7.63 | 0.221  (0.16)  31.13 | 0.02  (0.92)  1.42 | -0.17  (0.29)  6.75 | -0.04  (0.81)  17.06 | -0.03  (0.8)  4.26 |
| Sparkling white wine  Correlation coefficient (r)  P value  % contribution | 0.29  (0.07)  0.0 |  | 0.07  (0.68)  0.0 |  |  | 0.17  (0.30)  0.0 |
| Sherry  Correlation coefficient (r)  P value  % contribution | -0.08  (0.61)  0.01 |  | -0.17  (0.27)  0.01 |  |  | -0.09  (0.56)  0.0 |
| Blueberries  Correlation coefficient (r)  P value  % contribution | 0.19  (0.02)  1.08 | 0.15  (0.17)  7.94 | 0.24  (0.13)  0.06 | -0.04  (0.82)  0.30 |  | 0.30  (0.06)  0.61 |
| Blackberries  Correlation coefficient (r)  P value  % contribution | 0.55  (0.00)  0.02 | -0.02  (0.46)  0.42 | 0.56  (0.00)  0.01 |  |  | 0.40  (0.01)  0.01 |
| Strawberries  Correlation coefficient (r)  P value  % contribution | 0.13  (0.43)  0.61 | 0.102  (0.26)  5.5 | 0.09  (0.56)  0.16 | 0.28  (0.07)  0.14 | 0.104  (0.51)  0.0 | 0.33  (0.03)  0.30 |
| Raspberries  Correlation coefficient (r)  P value  % contribution | 0.20  (0.20)  0.18 | 0.24  (0.06)  1.42 | 0.16  (0.30)  0.03 |  |  | 0.28  (0.07)  0.06 |
| Red grapes  Correlation coefficient (r)  P value  % contribution | 0.39  (0.01)  2.08 | 0.27  (0.04)  13.29 | -0.09  0.58  (0.18) | 0.42  (0.01)  7.35 |  | 0.07  (0.65)  0.46 |
| Green grapes  Correlation coefficient (r)  P value  % contribution | 0.31  (0.04)  1.39 |  | 0.14  (0.38)  0.65 |  |  | 0.39  (0.01)  0.76 |
| Cherries  Correlation coefficient (r)  P value  % contribution | 0.58  (0.00)  1.20 | 0.41  (0.003)  10.28 | 0.05  (0.73)  0.94 |  |  | 0.36  (0.02) 1.21 |
| Grapefruit  Correlation coefficient (r)  P value  % contribution | 0.03  (0.83)  0.11 |  |  |  | 0.16  (0.32)  2.46 | 0.12  (0.45)  0.03 |
| Orange  Correlation coefficient (r)  P value  % contribution | 0.1  (0.05)  3.33 |  |  | 0.43  (0.004)  40.86 | 0.51  (0.001)  2.28 | 0.09  (0.57)  0.55 |
| Lemon  Correlation coefficient (r)  P value  % contribution | -0.11  (0.48)  0.27 |  | -0.11  (0.49)  0.0 | -0.04  (0.81)  2.26 | 0.16  (0.31)  1.17 | -0.11  (0.51)  0.10 |
| Lime  Correlation coefficient (r)  P value  % contribution | -0.02  (0.89)  0.01 |  |  |  | 0.04  (0.78)  0.22 | 0.02  (0.89)  0.0 |
| Mandarin  Correlation coefficient (r)  P value  % contribution | 0.2  (0.28)  2.07 |  |  |  | 0.55  (0.00)  19.18 |  |
| Pineapple  Correlation coefficient (r)  P value  % contribution | 0.11  (0.47)  0.00 |  |  | 0.69  (0.00)  0.0 |  | 0.27  (0.09)  0.03 |
| Rockmelon  Correlation coefficient (r)  P value  % contribution | -0.11  (0.48)  0.01 |  |  | -0.18  (0.25)  1.83 |  | -0.12  (0.49)  0.01 |
| Banana  Correlation coefficient (r)  P value  % contribution | -0.15  (0.35)  0.1 | -0.04  (0.39)  7.04 | -0.14  (0.39)  1.06 |  |  | -0.03  (0.84)  0.13 |
| Red apple  Correlation coefficient (r)  P value  % contribution | 0.3  (0.84)  1.86 | -0.09  (0.28)  3.78 | 0.03  (0.84)  2.48 | 0.08  (0.61)  0.05 |  | 0.13  (0.40)  3.37 |
| Green apple  Correlation coefficient (r)  P value  % contribution | 0.10  (0.53)  0.15 |  | 0.13  (0.43)  0.20 |  |  | 0.05  (0.76)  0.38 |
| Pear  Correlation coefficient (r)  P value  % contribution | 0.28  (0.07)  1.0 | -0.14  (0.19)  5.96 | 0.30  (0.03)  0.27 |  |  | 0.38  (0.01)  1.40 |
| Peach  Correlation coefficient (r)  P value  % contribution | 0.15  (0.34)  0.91 | 0.06  (0.36)  1.47 | 0.12  (0.45)  1.46 |  |  | 0.39  (0.01)  0.61 |
| Mango  Correlation coefficient (r)  P value  % contribution | 0.46  (0.002)  0.07 | 0.3  (0.03)  0.06 | 0.14  (0.37)  0.11 | 0.34  (0.03)  0.3 |  | 0.41  (0.01)  0.05 |
| Kiwi  Correlation coefficient (r)  P value  % contribution | -0.09  (0.59)  0.03 |  | -0.11  (0.50)  0.01 | 0.47  (0.002)  1.93 |  | -0.003  (0.98)  0.17 |
| Canned Peach  Correlation coefficient (r)  P value  % contribution | 0.18  (0.25)  0.03 |  | 0.21  (0.19)  0.07 |  |  |  |
| Plum  Correlation coefficient (r)  P value  % contribution | 0.02  (0.89)  0.40 | 0.08  (0.31)  2.72 | - | 0.17  (0.28)  0.11 |  | 0.28  (0.07)  0.63 |
| Nectarine  Correlation coefficient (r)  P value  % contribution | 0.54  (0.0)  0.70 | 0.41  (0.004)  1.39 | 0.08  (0.63)  1.02 |  |  | 0.32  (0.04)  0.54 |
| Fig  Correlation coefficient (r)  P value  % contribution | 0.28  (0.07)  0.01 | 0.12  (0.23)  0.0 | 0.25  (0.06)  0.0 |  |  | 0.45  (0.003)  0.17 |
| Tomato  Correlation coefficient (r)  P value  % contribution | 0.11  (0.51)  0.13 |  |  | -0.11  (0.49)  1.63 | 0.08  (0.60)  0.09 | 0.15  (0.35)  0.79 |
| Carrot  Correlation coefficient (r)  P value  % contribution | 0.05  (0.77)  0.03 |  |  | 0.01  (0.94)  0.89 |  | 0.10  (0.54)  0.22 |
| Lettuce  Correlation coefficient (r)  P value  % contribution | 0.22  (0.16)  0.06 |  |  | 0.14  (0.38)  1.08 |  | 0.32  (0.04)  0.52 |
| Rocket  Correlation coefficient (r)  P value  % contribution | 0.11  (0.51)  0.11 |  |  |  |  | 0.15  (0.34)  1.45 |
| Watercress  Correlation coefficient (r)  P value  % contribution | 0.34  (0.03)  0.0 |  |  | 0.26  (0.09)  0.0 |  | 0.53  (0.00)  0.06 |
| Silver beet  Correlation coefficient (r)  P value  % contribution | -0.07  (0.68)  0.29 |  | -0.07  (0.66)  0.15 |  |  | 0.15  (0.36)  1.61 |
| Endive  Correlation coefficient (r)  P value  % contribution | 0.42  (0.01)  0.0 |  |  |  |  | 0.31  (0.04)  0.0 |
| Radish  Correlation coefficient (r)  P value  % contribution | 0.003  (0.99)  0.06 | -0.02  (0.44)  0.65 |  |  |  | 0.24  (0.13)  0.02 |
| Cucumber  Correlation coefficient (r)  P value  % contribution | 0.15  (0.36)  0.01 |  |  | 0.09  (0.57)  0.01 |  | 0.25  (0.11)  0.11 |
| Celery  Correlation coefficient (r)  P value  % contribution | 0.36  (0.8)  2.0 |  |  | 0.10  (0.54)  4.02 |  | 0.26  (0.09)  0.05 |
| Avocado  Correlation coefficient (r)  P value  % contribution | 0.24  (0.13)  0.01 | 0.21  (0.09)  0.06 | 0.17  (0.28)  0.01 |  |  |  |
| Capsicum  Correlation coefficient (r)  P value  % contribution | 0.15  (0.36)  0.02 |  |  | 0.10  (0.52)  1.78 |  | 0.14  (0.38)  0.04 |
| Cabbage  Correlation coefficient (r)  P value  % contribution | -0.05  (0.76)  0.01 |  |  | 0.12  (0.46)  0.61 |  | -0.13  (0.42)  0.10 |
| Beans  Correlation coefficient (r)  P value  % contribution | 0.1  (0.53)  1.0 | -0.02  (0.45)  1.36 |  |  |  | 0.24  (0.12)  8.93 |
| Broccoli  Correlation coefficient (r)  P value  % contribution | 0.16  (0.31)  2.57 |  | 0.12  (0.43)  4.80 |  |  | 0.14  (0.39)  1.94 |
| Peas  Correlation coefficient (r)  P value  % contribution | 0.13  (0.41)  1.1 |  | 0.25  (0.12)  5.04 |  |  | 0.12  (0.46)  0.45 |
| Zucchini  Correlation coefficient (r)  P value  % contribution | 0.07  (0.68)  0.01 |  |  |  |  | 0.22  (0.17)  0.08 |
| Eggplant  Correlation coefficient (r)  P value  % contribution | 0.09  (0.07)  0.09 | 0.24  (0.06)  1.11 |  |  |  | 0.31  (0.05)  0.0 |
| Brussels  Correlation coefficient (r)  P value  % contribution | 0.04  (0.80)  0.08 |  |  | -0.08  (0.63)  3.59 | -0.27  (0.09)  0.89 | 0.15  (0.33)  0.40 |
| Spinach  Correlation coefficient (r)  P value  % contribution | 0.28  (0.07)  1.33 |  |  | 0.33  (0.03)  2.30 |  | 0.42  (0.01)  2.60 |
| Kale  Correlation coefficient (r)  P value  % contribution | 0.45  (0.003)  1.24 |  |  |  |  | 0.43  (0.005)  2.66 |
| Asparagus  Correlation coefficient (r)  P value  % contribution | -0.006  (0.97)  0.04 |  |  |  |  | 0.03  (0.87)  0.37 |
| Potato  Correlation coefficient (r)  P value  % contribution | 0.06  (0.69)  0.12 |  |  | -0.04  (0.77)  0.0 |  | 0.16  (0.31)  1.36 |
| Pumpkin  Correlation coefficient (r)  P value  % contribution | -0.11  (0.48)  0.09 |  |  | 0.09  (0.56)  11.08 |  |  |
| Brown onion  Correlation coefficient (r)  P value  % contribution | 0.22  (0.16)  1.09 |  |  |  |  | 0.54  (0.00)  9.79 |
| Red onion  Correlation coefficient (r)  P value  % contribution | 0.04  (0.80)  1.6 | -0.06  (0.35)  0.93 |  | -0.04  (0.77)  0.97 |  | 0.26  (0.10)  4.44 |
| Spring onion  Correlation coefficient (r)  P value  % contribution | 0.13  (0.43)  0.10 |  |  |  |  | 0.44  (0.004)  1.06 |
| Cauliflower  Correlation coefficient (r)  P value  % contribution | 0.06  (0.70)  0.03 |  |  | 0.15  (0.33)  1.17 |  | 0.07  (0.65)  0.22 |
| Parsnip  Correlation coefficient (r)  P value  % contribution | -0.13  (0.40)  0.01 |  |  |  |  | -0.06  (0.73)  0.04 |
| Capers  Correlation coefficient (r)  P value  % contribution | -0.06  (0.71)  0.06 |  |  |  |  | 0.24  (0.13)  0.75 |
| Parsley  Correlation coefficient (r)  P value  % contribution | 0.25  (0.11)  1.0 |  |  | 0.98  (0.00)  35.50 |  | 0.15  (0.35)  0.45 |
| Dill  Correlation coefficient (r)  P value  % contribution | 0.38  (0.01)  0.0 |  |  |  |  | 0.29  (0.06)  0.03 |
| Ginger  Correlation coefficient (r)  P value  % contribution | 0.002  (0.99)  0.06 |  |  |  |  | -0.09  (0.58)  0.87 |
| Garlic  Correlation coefficient (r)  P value  % contribution | 0.13  (0.41)  0.01 |  |  |  |  | 0.15  (0.35)  0.07 |
| Sultana/raisins  Correlation coefficient (r)  P value  % contribution | 0.39  (0.01)  1.0 | 0.30  (0.03)  0.01 | 0.38  (0.01)  0.01 | -0.11  (0.48)  0.04 |  | 0.44  (0.003)  0.03 |
| Date  Correlation coefficient (r)  P value  % contribution | -0.06  (0.71)  0.02 | -0.18  (0.51)  0.26 |  |  |  | 0.09  (0.58)  0.09 |
| Dark chocolate  Correlation coefficient (r)  P value  % contribution | -0.10  (0.52)  0.2 |  | 0.04  (0.78)  0.35 |  |  |  |
| Milk Chocolate  Correlation coefficient (r)  P value  % contribution | 0.14  (0.37)  0.13 |  | -0.23  (0.14)  0.34 |  |  |  |
| Chocolate biscuit  Correlation coefficient (r)  P value  % contribution | -0.18  (0.26)  0.05 |  | -0.10  (0.55)  0.11 |  |  |  |
| Marmalade  Correlation coefficient (r)  P value  % contribution | 0.04  (0.81)  0.02 |  |  |  | 0.06  (0.70)  0.60 |  |
| Jam  Correlation coefficient (r)  P value  % contribution | 0.05  (0.77)  0.0 | -0.15  (0.17)  0.0 | 0.15  (0.34)  0.0 |  |  | 0.23  (0.14)  0.0 |
| Berry Jam  Correlation coefficient (r)  P value  % contribution | -0.09  (0.56)  0.02 |  | -0.03  (0.87)  0.04 |  |  | -0.09  (0.56)  0.12 |
| Honey  Correlation coefficient (r)  P value  % contribution | -0.08  (0.63)  0.01 |  |  | -0.01  (0.96)  0.35 |  | -0.04  (0.79)  0.05 |

Appendix 1.- FlavQ

**Instructions For Completing The
Flavonoid Food Frequency Questionnaire (Flav-Q)**

**What is a FFQ?**

Dietary flavonoids are a group of natural, plant-based compounds that often provide the colour in the fruits and vegetables we consume. The Flav-Q is a survey for estimating your intake of several foods over the past year, which should help us understand your usual intake of flavonoids.

**What all will I be asked?**

The FFQ usually takes about 15 minutes to complete. You will be asked how often you eat some important flavonoid rich foods including a few summary questions about your overall fruit and vegetable intake.

**How often?**

For each item, choose the one best “how often” answer for you that best describes your intake on average over the whole year. Your options will range from never to a specific number of times per month, week or day.

**How much?**

Portion sizes for each food are listed. Choose the closest answer to your usual portion size.

**Thank you for taking the time to complete this survey.**

**First we will start with some questions about you:**

**Date of Birth** *(we will use this to determine your age)*

______________________________
**Post-code** *(we will use this to determine if you live in a rural or urban location)*

_________________________________

**Gender**

Male Female Other _____________

**What is your HEIGHT (in cm)** *(if unsure, please estimate to the best of your ability)*

**_______________**

**What is your WEIGHT (in kg)** *(if unsure, please estimate to the best of your ability)*

**_______________**

**Primary Language -** *What is the primary language you speak at home?*

English Other _______________________

**Education -** *What is the highest level of education you obtained?*

Primary School Secondary School Tertiary (University or TAFE)

**Smoking Status**

Never Smoker Current Smoker Previously a Smoker

*If you are, or were previously a smoker please provide details on the extent of your smoking habits:*

2x pack/day or more 1x pack/day Less than 1xpack/day

**Alcohol Intake**

Don’t drink alcohol Rarely Monthly Weekly Daily

**Regular Supplement Use**

*Supplements are a product intended to complement the*[*diet*](http://en.wikipedia.org/wiki/Diet_(nutrition))*and may contain a*[*vitamin*](http://en.wikipedia.org/wiki/Vitamin)*, a*[*mineral*](http://en.wikipedia.org/wiki/Dietary_mineral) *or other*[*botanical*](http://en.wikipedia.org/wiki/Botanical) (*e.g. Fish oil, Multivitamin, Glucosamine).*

*What supplements do you take?*

Do not take supplements Take supplements (please list names/brands)

______________________________________________

**How many pieces of fruit do you usually eat per day?**

*(Count 1 medium apple or 1/2 cup of diced fruit, berries or grapes as one piece)*

- None
- less than 1 piece of fruit per day
- 1 piece of fruit per day
- 2 pieces of fruit per day
- 3 pieces of fruit per day
- 4 or more pieces of fruit per day

**How many serves of vegetables do you usually eat per day?**

*(Count all types, fresh, frozen or tinned. Count 1 medium potato, or ½ cup cooked or 1 cup fresh vegetables as one serve)*

- None
- less than 1 vegetable per day
- 1 vegetable per day
- 2 vegetables per day
- 3 vegetables per day
- 4 vegetables per day
- 5 vegetables per day
- 6 or more vegetables per day

|  | **Over the past 12 months, how often did you consume the following foods…** | | | | | | | | | | | | | | | | | **How much?** | | | | | |
| --- | --- | --- | --- | --- | --- | --- | --- | --- | --- | --- | --- | --- | --- | --- | --- | --- | --- | --- | --- | --- | --- | --- | --- |
|  | *Please tick the frequency option that best corresponds with your intake* | | | | | | | | | | | | | | | | | *Please tick the portion size which best corresponds with your intake* | | | | | |
|  | **Never** | **per month** | | | | **per week** | | | | | | **per day** | | | | | |  |  |  |  |  |  |
|  |  | **1 time or less** | | **2 to 3 times** | | **1 to 2 times** | | **3 to 4 times** | | **5 to 6 times** | | **1 time** | | **2 to 3 times** | | **4 or more times** | |  |  |  |  |  |  |
| **Beverages** | | | | | | | | | | | | | | | | | | | | | | | |
| Black Tea | ○ | | ○ | | ○ | | ○ | | ○ | | ○ | | ○ | | ○ | | ○ | Less than a small cup (<180ml) | | A small cup (180ml) | An average cup (250ml) | | More than a cup (>250ml) |
|  |  |  |  |  |  |  |  |  |  |  |  |  |  |  |  |  |  | ○ | | ○ | ○ | | ○ |
| Green Tea | ○ | | ○ | | ○ | | ○ | | ○ | | ○ | | ○ | | ○ | | ○ | Less than a small cup (<180ml) | | A small cup (180ml) | An average cup (250ml) | | More than a cup (>250ml) |
|  |  |  |  |  |  |  |  |  |  |  |  |  |  |  |  |  |  | ○ | | ○ | ○ | | ○ |
| Orange Juice | ○ | | ○ | | ○ | | ○ | | ○ | | ○ | | ○ | | ○ | | ○ | Less than 1 glass (<150ml) | | 1 glass (150ml) | 2 glasses (300ml) | | More than 2 glasses (>300ml) |
|  |  |  |  |  |  |  |  |  |  |  |  |  |  |  |  |  |  | ○ | | ○ | ○ | | ○ |
| Blackcurrant Juice | ○ | | ○ | | ○ | | ○ | | ○ | | ○ | | ○ | | ○ | | ○ | Less than 1 glass (<150ml) | | 1 glass (150ml) | 2 glasses (300ml) | | More than 2 glasses (>300ml) |
|  |  |  |  |  |  |  |  |  |  |  |  |  |  |  |  |  |  | ○ | | ○ | ○ | | ○ |
| Red Wine | ○ | | ○ | | ○ | | ○ | | ○ | | ○ | | ○ | | ○ | | ○ | Less than 1 glass (<150ml) | | 1 glass (150ml) | 2 glasses (300ml) | | More than 2 glasses (>300ml) |
|  |  |  |  |  |  |  |  |  |  |  |  |  |  |  |  |  |  | ○ | | ○ | ○ | | ○ |
| **Fruit** *(including fresh, frozen or tinned fruits. For seasonal fruit, please describe your intake when the food is in season only)* | | | | | | | | | | | | | | | | | | | | | | | |
| Blueberries | ○ | | ○ | | ○ | | ○ | | ○ | | ○ | | ○ | | ○ | | ○ | Less than ¼ cup | | ¼ a cup | ½ a cup | | More than ½ a cup |
|  |  |  |  |  |  |  |  |  |  |  |  |  |  |  |  |  |  | ○ | | ○ | ○ | | ○ |
| Red Grapes | ○ | | ○ | | ○ | | ○ | | ○ | | ○ | | ○ | | ○ | | ○ | Less than ½ cup | | ½ a cup (around 15 grapes) | 1 cup (around 30 grapes) | | More than 1 cup |
|  |  |  |  |  |  |  |  |  |  |  |  |  |  |  |  |  |  | ○ | | ○ | ○ | | ○ |
| Green Grapes | ○ | | ○ | | ○ | | ○ | | ○ | | ○ | | ○ | | ○ | | ○ | Less than ½ cup | | ½ a cup (around 15 grapes) | 1 cup (around 30 grapes) | | More than 1 cup |
|  |  |  |  |  |  |  |  |  |  |  |  |  |  |  |  |  |  | ○ | | ○ | ○ | | ○ |
| Cherries | ○ | | ○ | | ○ | | ○ | | ○ | | ○ | | ○ | | ○ | | ○ | Less than ½ cup | | ½ a cup | 1 cup | | More than 1 cup |
|  |  |  |  |  |  |  |  |  |  |  |  |  |  |  |  |  |  | ○ | | ○ | ○ | | ○ |
| Oranges | ○ | | ○ | | ○ | | ○ | | ○ | | ○ | | ○ | | ○ | | ○ | Less than 1 small orange | | ½ a medium orange OR 1 small orange | 1 medium orange | | More than 1 medium orange |
|  |  |  |  |  |  |  |  |  |  |  |  |  |  |  |  |  |  | ○ | | ○ | ○ | | ○ |
|  | **Over the past 12 months, how often did you consume the following foods…** | | | | | | | | | | | | | | | | | **How much?** | | | | | |
|  | *Please tick the frequency option that best corresponds with your intake* | | | | | | | | | | | | | | | | | *Please tick the portion size which best corresponds with your intake* | | | | | |
|  | **Never** | **per month** | | | | **per week** | | | | | | **per day** | | | | | |  |  |  |  |  |  |
|  |  | **1 time or less** | | **2 to 3 times** | | **1 to 2 times** | | **3 to 4 times** | | **5 to 6 times** | | **1 time** | | **2 to 3 times** | | **4 or more times** | |  |  |  |  |  |  |
| Mandarins | ○ | | ○ | | ○ | | ○ | | ○ | | ○ | | ○ | | ○ | | ○ | Less than 1 small mandarin | 1 small mandarin | | 1 large OR 2 small mandarin | More than 1 large OR 2 small mandarin | |
|  |  |  |  |  |  |  |  |  |  |  |  |  |  |  |  |  |  | ○ | ○ | | ○ | ○ | |
| Red Apples | ○ | | ○ | | ○ | | ○ | | ○ | | ○ | | ○ | | ○ | | ○ | Less than 1 small apple | 1 small apple | | 1 large apple | More than 1 large apple | |
|  |  |  |  |  |  |  |  |  |  |  |  |  |  |  |  |  |  | ○ | ○ | | ○ | ○ | |
| Pears | ○ | | ○ | | ○ | | ○ | | ○ | | ○ | | ○ | | ○ | | ○ | Less than 1 small pear | 1 small pear | | 1 large pear | More than 1 large pear | |
|  |  |  |  |  |  |  |  |  |  |  |  |  |  |  |  |  |  | ○ | ○ | | ○ | ○ | |
| Sultanas/Raisins | ○ | | ○ | | ○ | | ○ | | ○ | | ○ | | ○ | | ○ | | ○ | Less than ¼ cup | ¼ cup (30g) | | 1/3 cup (45g) | More than 1/3 cup  ○ | |
|  |  |  |  |  |  |  |  |  |  |  |  |  |  |  |  |  |  | ○ | ○ | | ○ |  |  |
| **Vegetables** *(including fresh, cooked, frozen or tinned)* | | | | | | | | | | | | | | | | | | | | | | | |
| Beans | ○ | | ○ | | ○ | | ○ | | ○ | | ○ | | ○ | | ○ | | ○ | Less than ½ cup | | ½ a cup | 1 cup | | More than 1 cup |
|  |  |  |  |  |  |  |  |  |  |  |  |  |  |  |  |  |  | ○ | | ○ | ○ | | ○ |
| Broccoli | ○ | | ○ | | ○ | | ○ | | ○ | | ○ | | ○ | | ○ | | ○ | Less than ½ cup | | ½ a cup | 1 cup | | More than 1 cup |
|  |  |  |  |  |  |  |  |  |  |  |  |  |  |  |  |  |  | ○ | | ○ | ○ | | ○ |
| Peas | ○ | | ○ | | ○ | | ○ | | ○ | | ○ | | ○ | | ○ | | ○ | Less than ½ cup | | ½ a cup | 1 cup | | More than 1 cup |
|  |  |  |  |  |  |  |  |  |  |  |  |  |  |  |  |  |  | ○ | | ○ | ○ | | ○ |
| Kale | ○ | | ○ | | ○ | | ○ | | ○ | | ○ | | ○ | | ○ | | ○ | Less than ½ cup | | ½ a cup | 1 cup | | More than 1 cup |
|  |  |  |  |  |  |  |  |  |  |  |  |  |  |  |  |  |  | ○ | | ○ | ○ | | ○ |
| Celery | ○ | | ○ | | ○ | | ○ | | ○ | | ○ | | ○ | | ○ | | ○ | Less than ½ cup | | ½ a cup | 1 cup | | More than 1 cup |
|  |  |  |  |  |  |  |  |  |  |  |  |  |  |  |  |  |  | ○ | | ○ | ○ | | ○ |
| Spinach | ○ | | ○ | | ○ | | ○ | | ○ | | ○ | | ○ | | ○ | | ○ | Less than ½ cup | | ½ a cup | 1 cup | | More than 1 cup |
|  |  |  |  |  |  |  |  |  |  |  |  |  |  |  |  |  |  | ○ | | ○ | ○ | | ○ |
| Brown onion | ○ | | ○ | | ○ | | ○ | | ○ | | ○ | | ○ | | ○ | | ○ | Less than ½ cup | | ½ a cup | 1 cup | | More than 1 cup |
|  |  |  |  |  |  |  |  |  |  |  |  |  |  |  |  |  |  | ○ | | ○ | ○ | | ○ |
| Red Onion | ○ | | ○ | | ○ | | ○ | | ○ | | ○ | | ○ | | ○ | | ○ | Less than ½ cup | | ½ a cup | 1 cup | | More than 1 cup |
|  |  |  |  |  |  |  |  |  |  |  |  |  |  |  |  |  |  | ○ | | ○ | ○ | | ○ |
| Parsley | ○ | | ○ | | ○ | | ○ | | ○ | | ○ | | ○ | | ○ | | ○ | Less than 1 tablespoon | | 1 tablespoon | ¼ cup | | More than ¼ cup |
|  |  |  |  |  |  |  |  |  |  |  |  |  |  |  |  |  |  | ○ | | ○ | ○ | | ○ |

Appendix 2- Lifestyle and eating


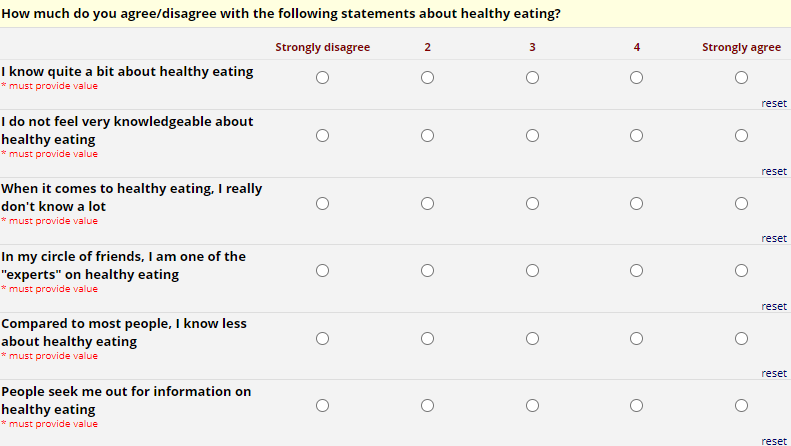

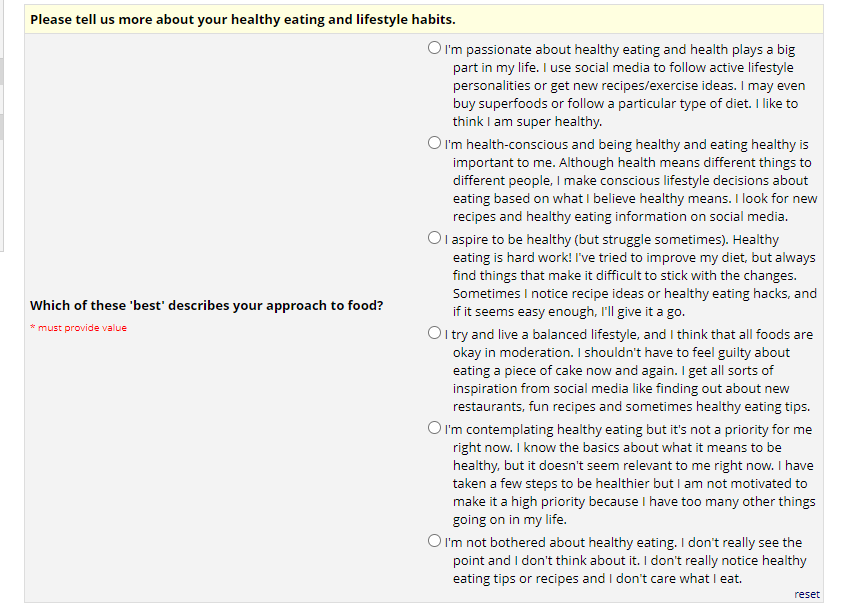


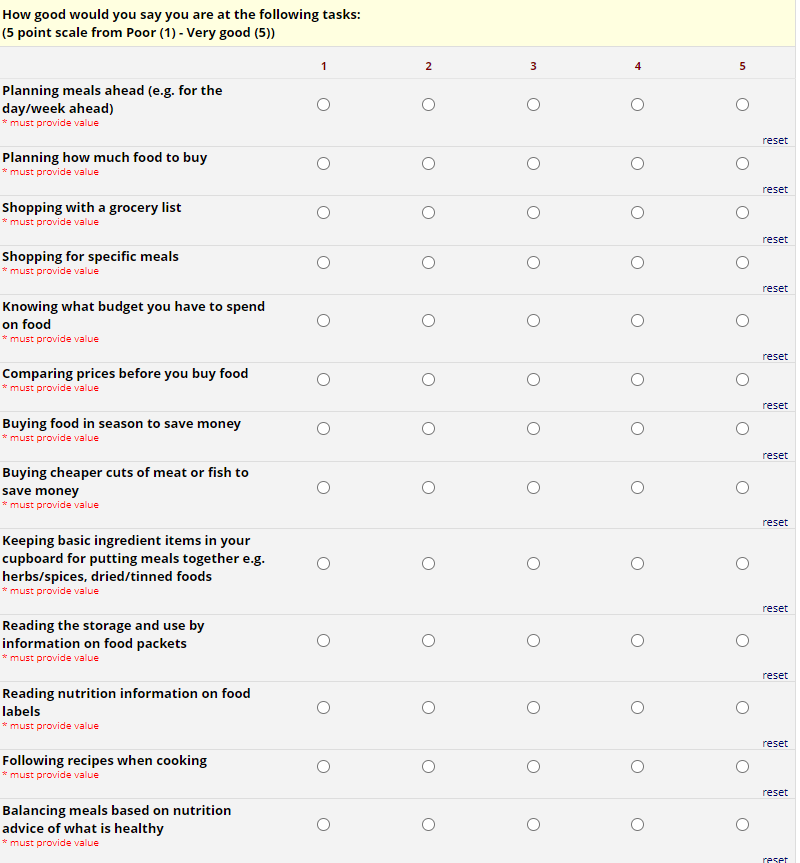


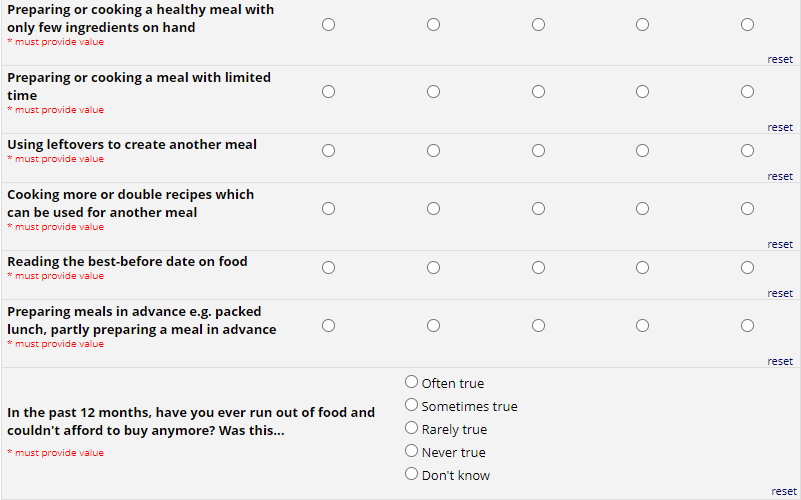


Appendix 3- Intake24 tool example

We are used the software Intake24 to measure participants’ dietary intake. This software asks participants to recall everything they have eaten in the last 24 hours using a multi-step process. Please see the attached images (with the example of entering muesli and coffee for breakfast).


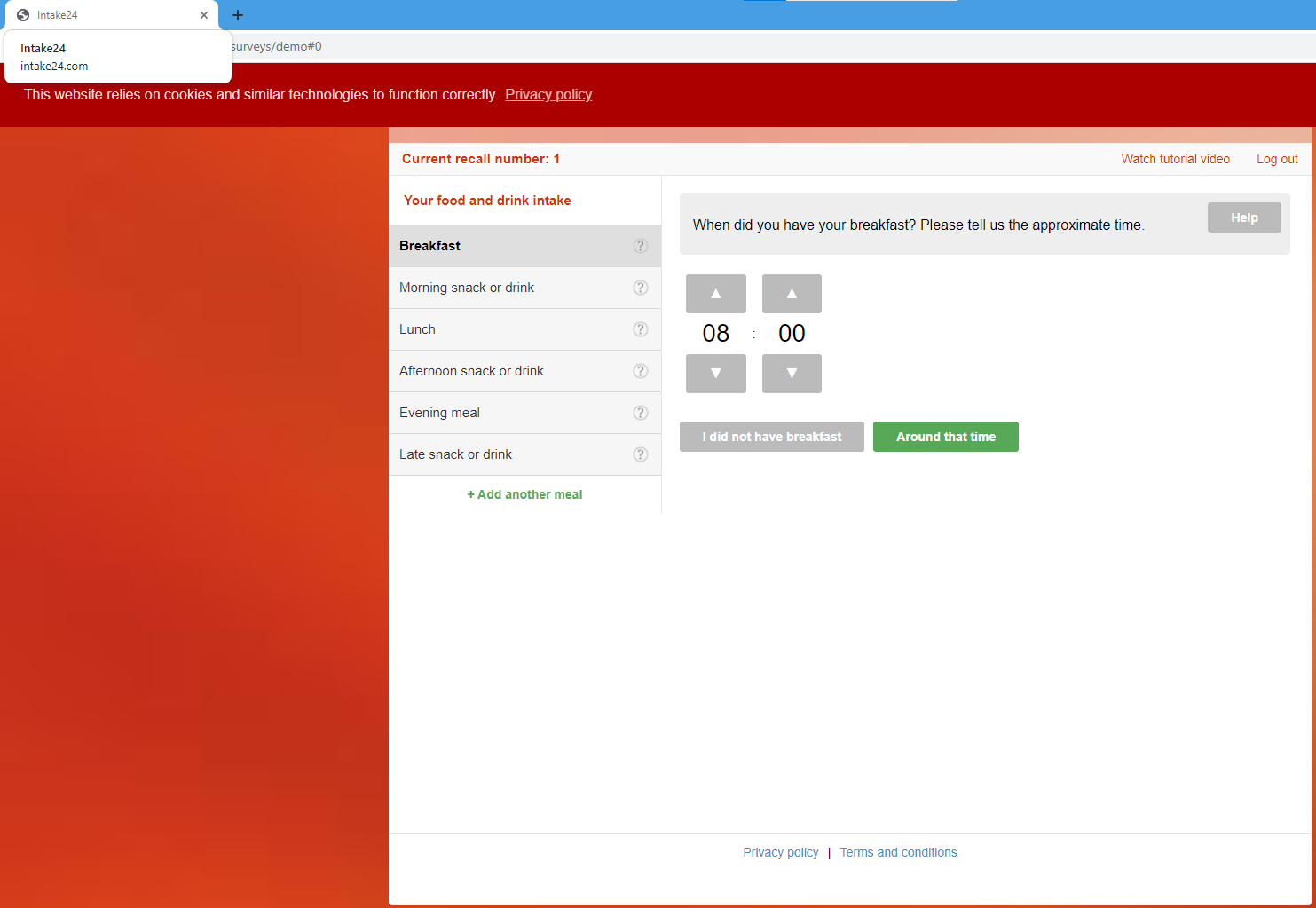

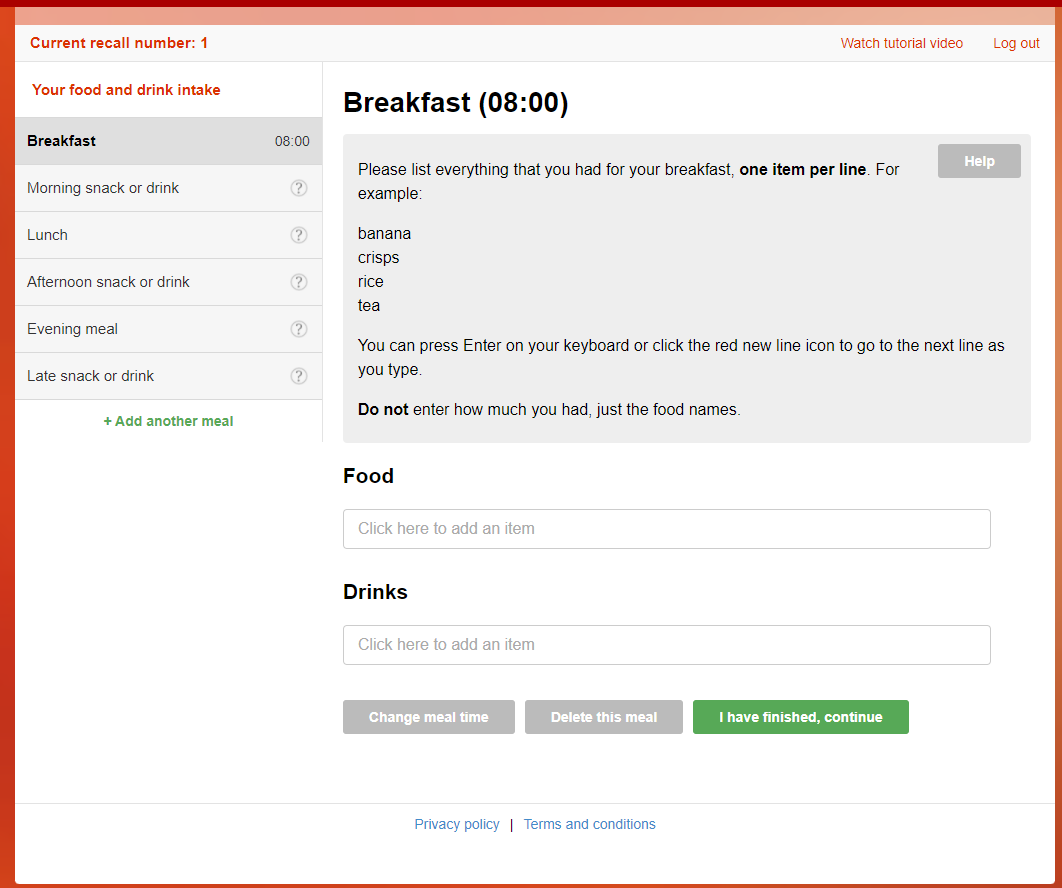

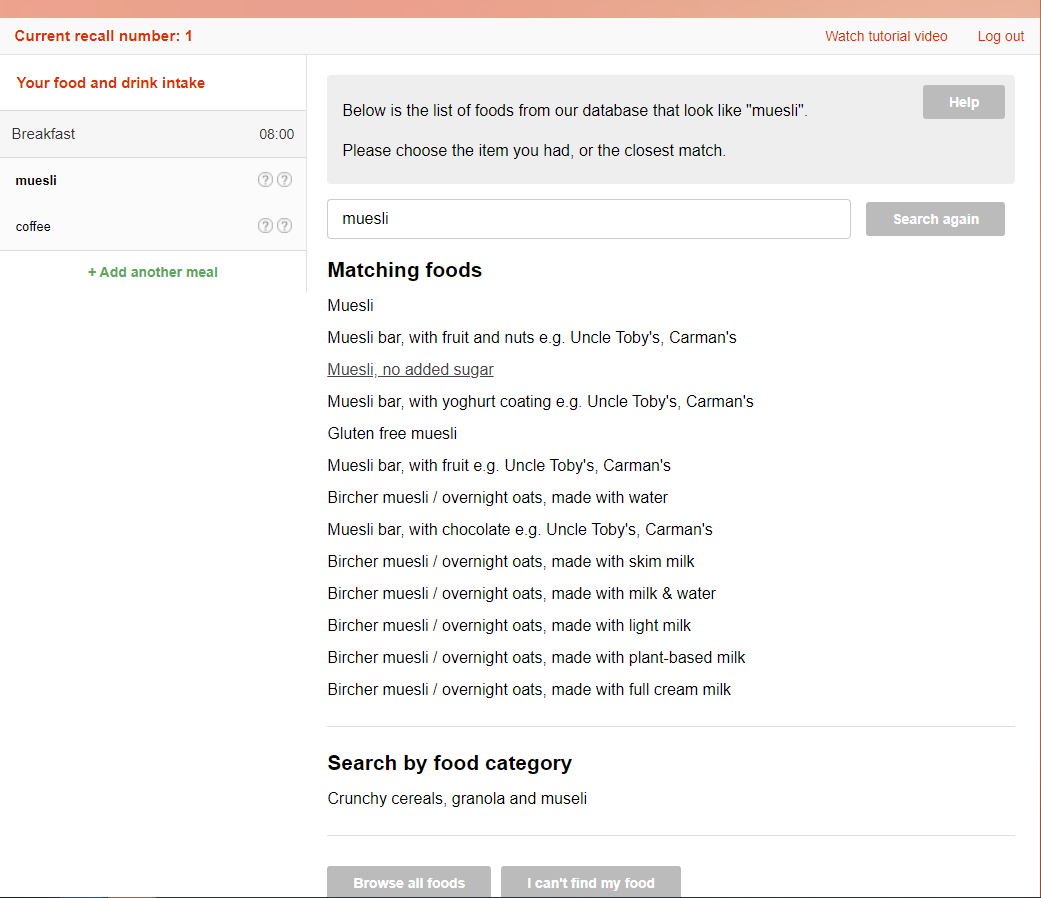

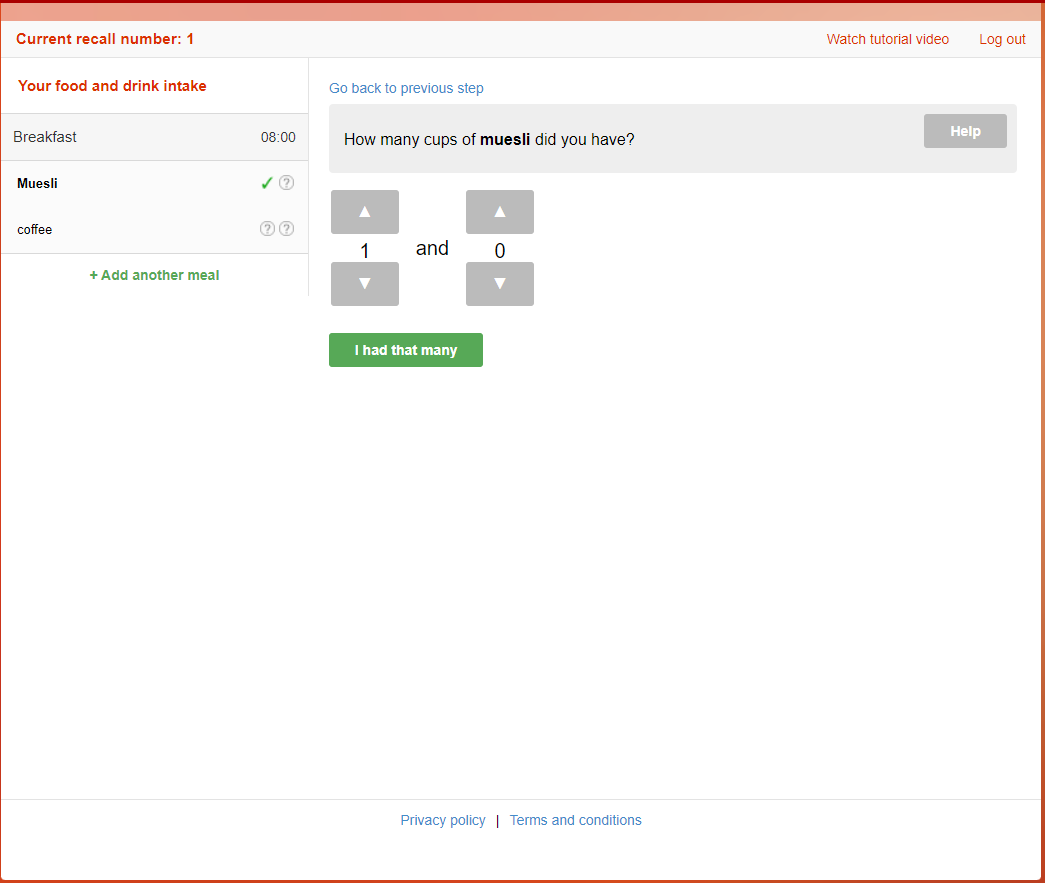


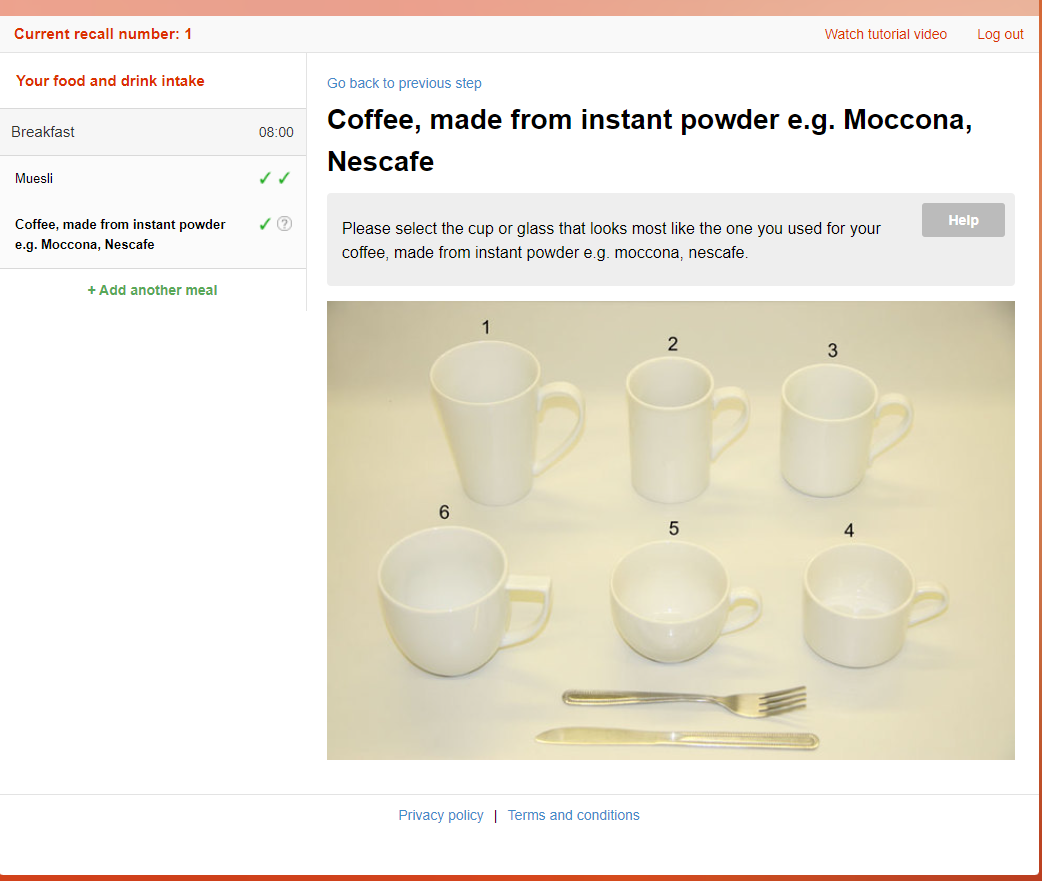


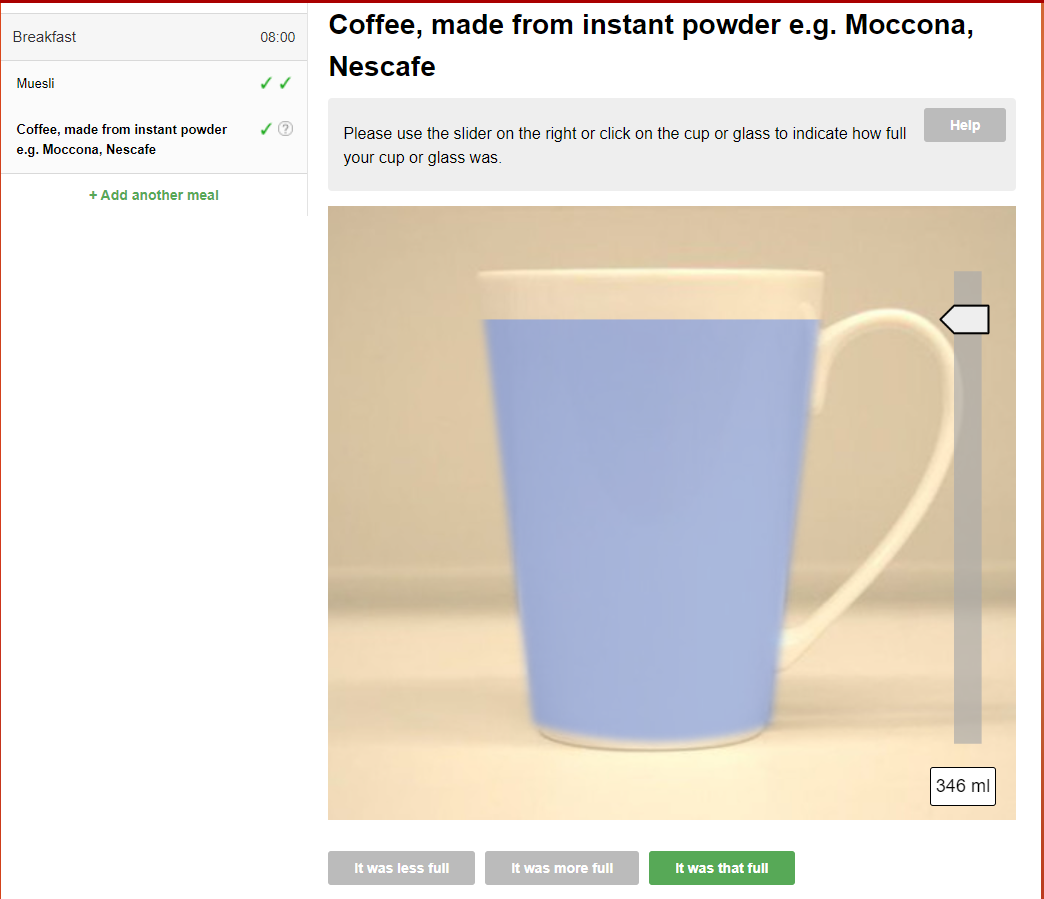


Appendix 4

Bland-altman plots for reproducibility

The bias is high to moderate with wide limits of agreement, suggesting overestimation by FLAV-Q1 for total flavonoid (mean bias= +79.76 mg/day, 95% CI= -390.12, 549.64, Bland-Altman index: 3.8%), flavan3ols (mean value = +41.45 mg/day, 95% CI= -299.83, 382.73, Bland-Altman index: 5%), anthocyanin intake (mean bias: +20.42 mg/day, 95% CI= -182.86, 223.7, Bland-Altman index: 3.8%) and flavonol intake (mean bias: +18.25 mg/day, 95% CI= -86.67, 123.17, Bland-Altman index: 5%). Narrow limits of agreement with small bias were reported for flavone (mean bias = 0.63, 95% CI= -67.76, 8.03, Bland-Altman index: 3.8%) and flavanone intake (mean bias: -0.03, 95% CI= -49.09, 49.02, Bland-Altman index: 2.5%). The linear correlation according to the Bland-Altman plots between FLAV-Q1 and FLAV-Q2 is weak for total flavonoid (R^2^ = 0.17), flavan3ol (R^2^= 0.11), flavanone (R^2^= 0.06) and flavone (R^2^= 0.25) showing no systematic increase in the intake by FLAV-Q1. The moderate linear regression for anthocyanin (R^2^= 0.36), flavonol (R^2^= 0.34) suggest that the trend in increase is systematic, showing better agreement for these two subclasses in lower intakes.

Further illustration of Bland-Altman plots for timepoint 1 and 3 and 1 and four is presented in Supplementary file 1. The substantial bias and wide limits of agreement between FLAV-Q1 and FLAV-Q3 has been shown particularly for total flavonoid intake (mean bias = +96.34 mg/day, 95% CI= -409.54, 602.22, Bland-Altman index: 2.5%), though the bias decreases for other flavonoid subclasses. The correlation between these two measurements is generally poor, indicating inconsistent bias with intake. Lastly, FLAV-Q1 and FLAV-Q4 show moderate bias for total flavonoid (mean bias = 82.67, 95% CI = -486.19, 651.54, Bland-Altman index: 2.5%) and flavan-3-ol intake (mean bias = 50.16, 95% CI = -374.04, 474.36, Bland-Altman: 2.4%), with smaller biases for other subclasses. Linear regression between FLAV-Q1 and FLAV-Q4 indicates more consistent agreement, particularly at the baseline (FLAV-Q1) and end of the study (FLAV-Q4).

| 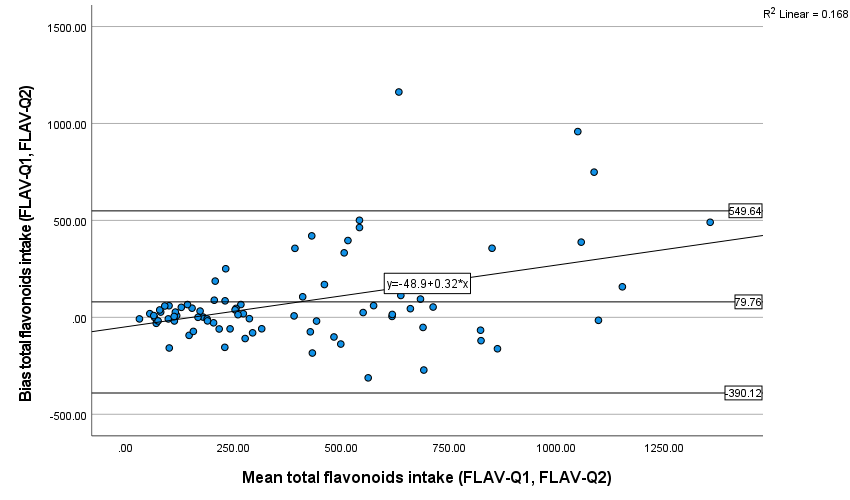  A) | 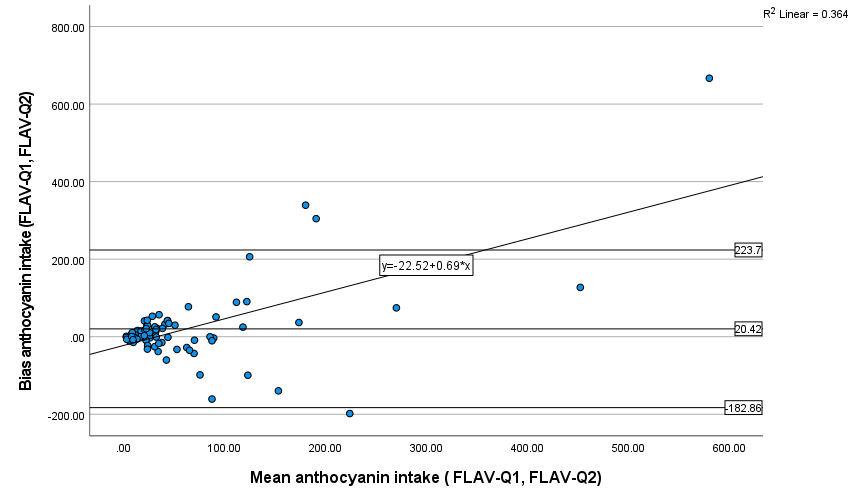  B) |
| --- | --- |
| 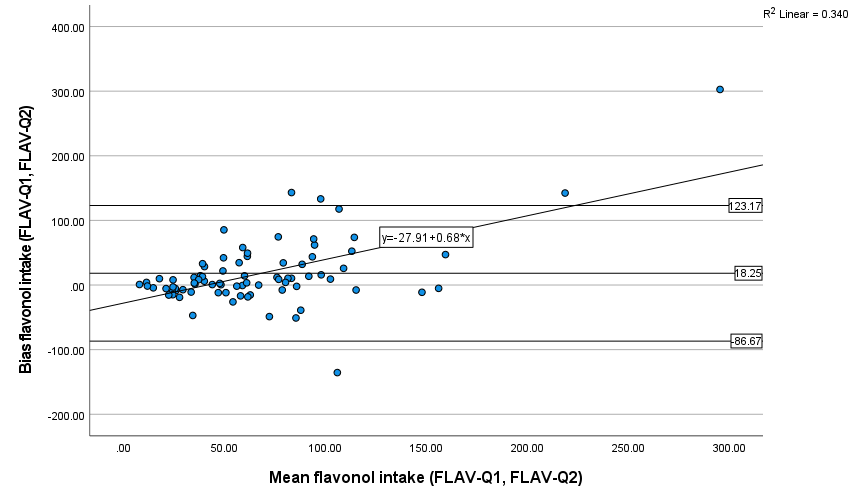  C)  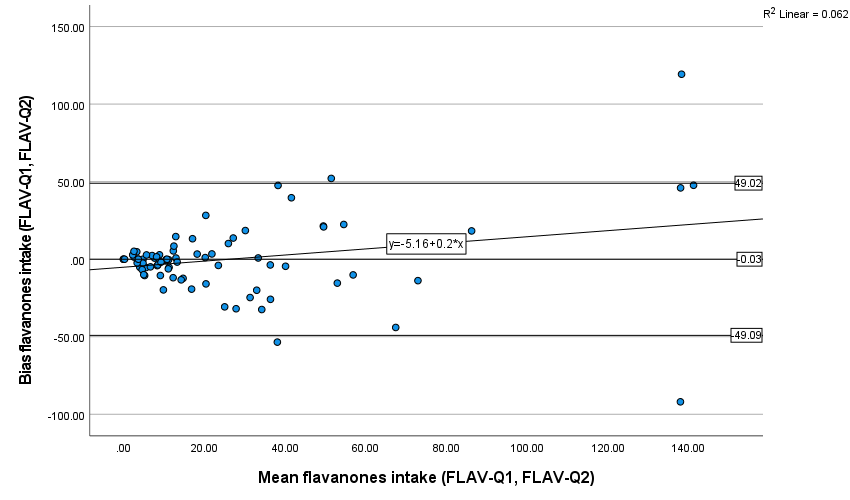  E) | 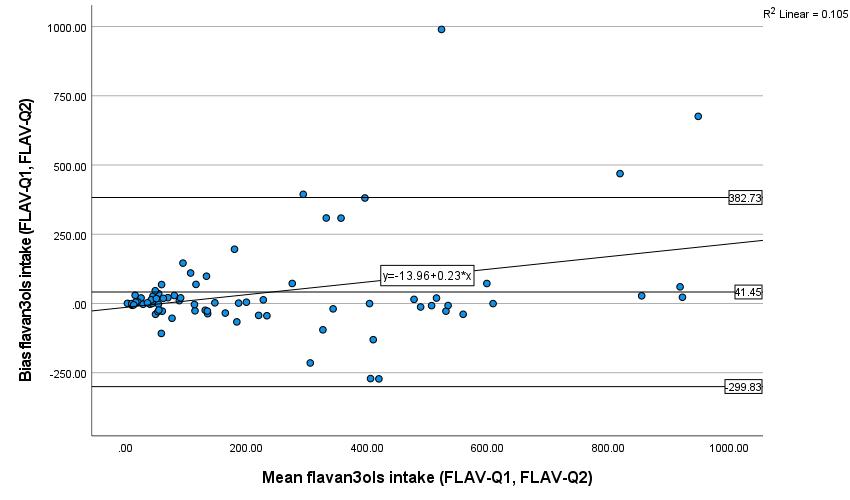  D)  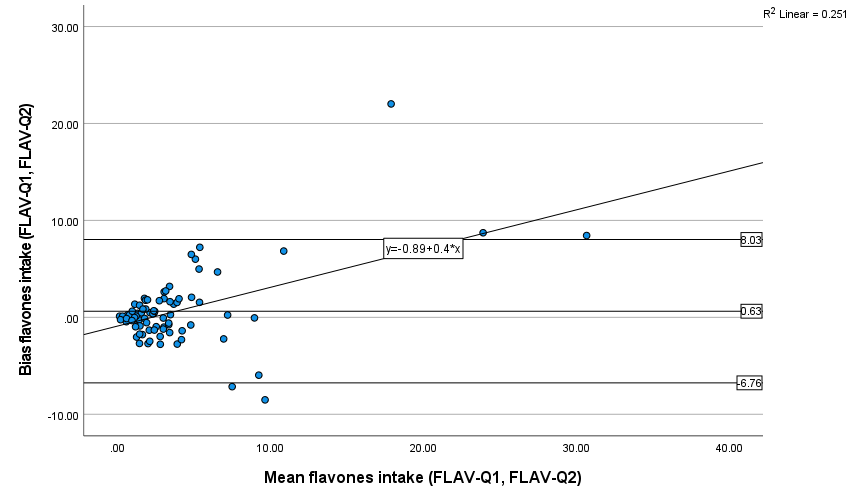  F) |

Fig.3 Bland-Altman plots (difference in intake (mg/day) (FLAV-Q1 – FLAV-Q2) against the mean intake of flavonoids and subclasses (mg/day) [FLAV-Q1 + FLAV-Q2)/2]) showing the relative validity of the FLAV-Q1 versus the FLAV-Q2 (A) flavonoids, (B) anthocyanin, (C) flavonol, (D) flavan3ol, (E) flavanone and (F) flavone intake

Overall, the Bland-Altman analysis reveals that while there is a generally moderate to small bias and reasonable agreement between the FLAV-Qs at different time points, the limits of agreement vary across different flavonoid subclasses. The narrowest limits of agreement were observed for flavones, indicating a higher level of consistency between FLAV-Q measurements for this subclass. Conversely, the declining correlation at each timepoints suggests that the agreement between FLAV-Q1 and subsequent FLAV-Qs (FLAV-Q2, FLAV-Q3 and FLAV-Q4) decreases over time. Variation increases at higher intakes and slopes are influenced by higher outlying values.

| 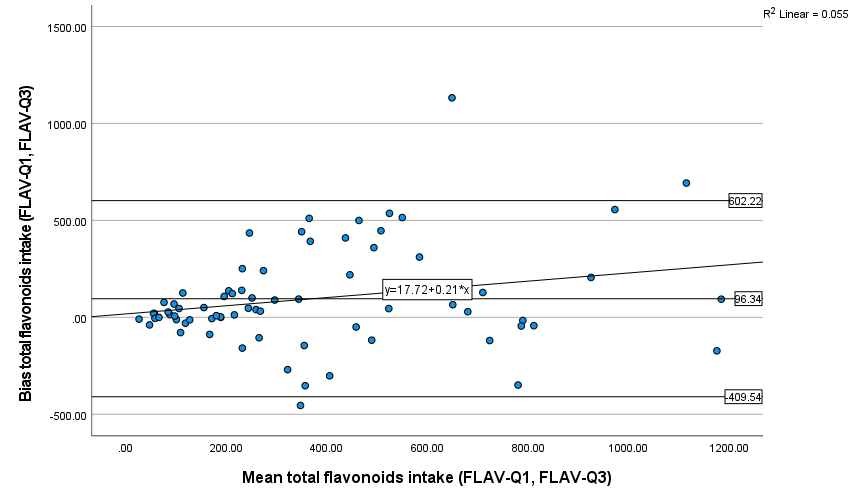 | 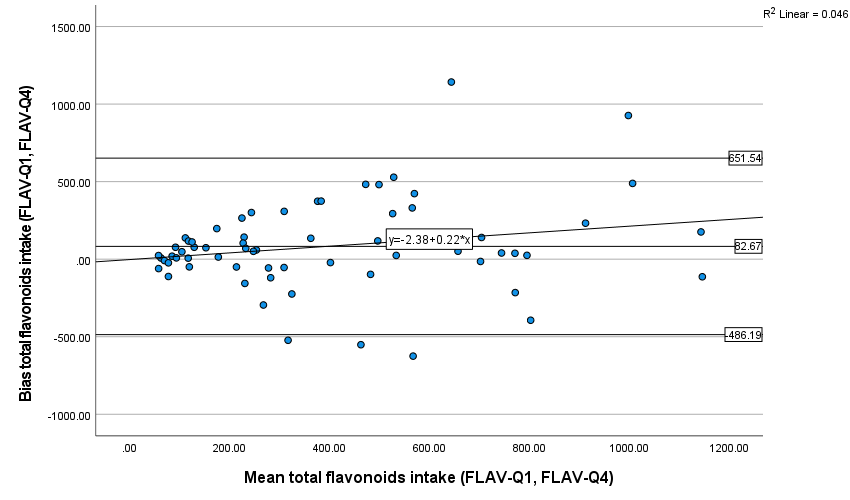 |
| --- | --- |
| 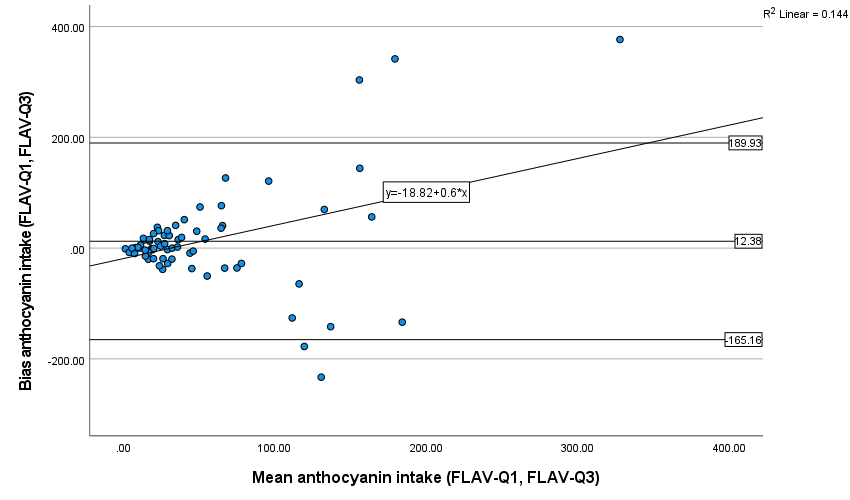 | 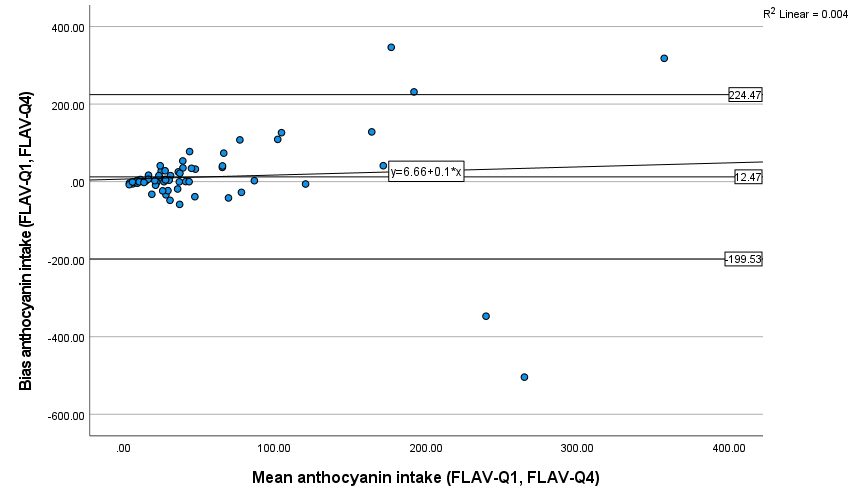 |
| 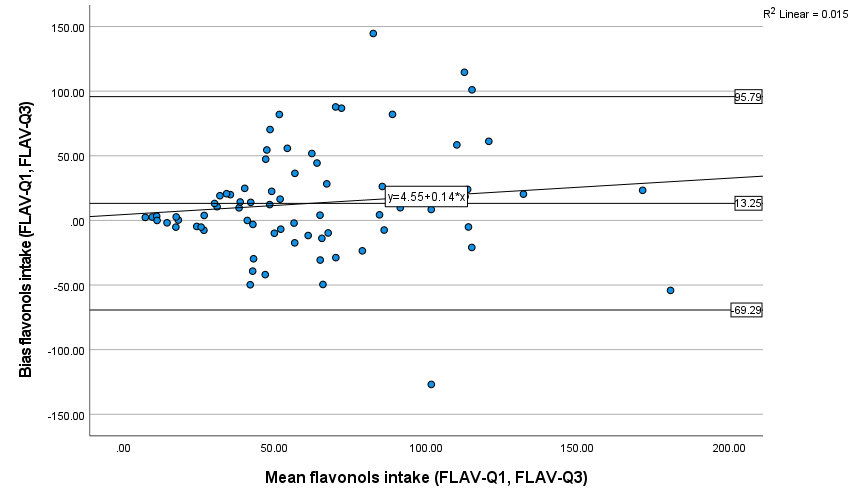 | 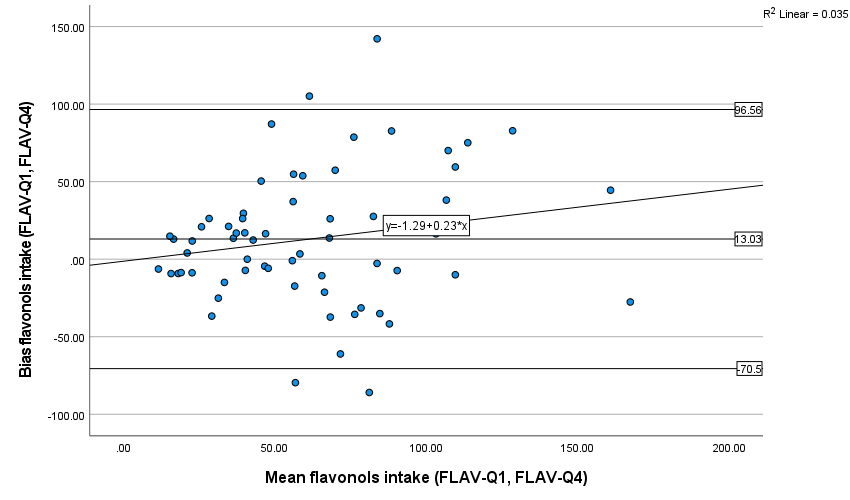 |
| 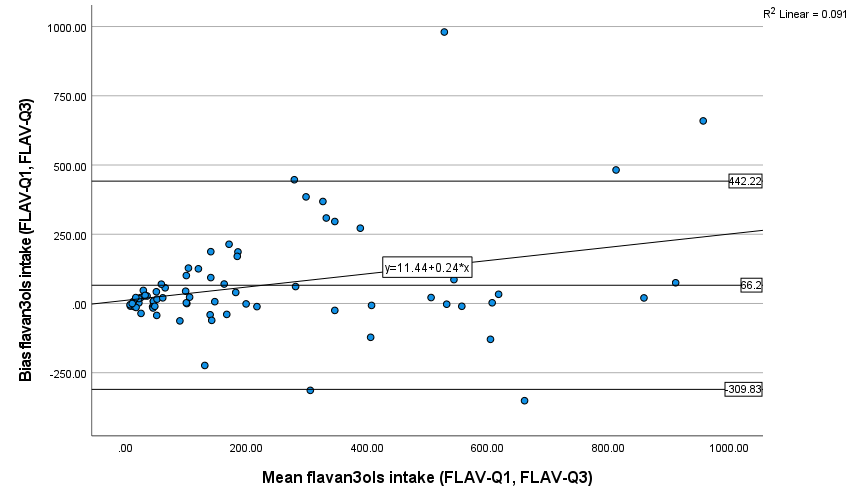 | 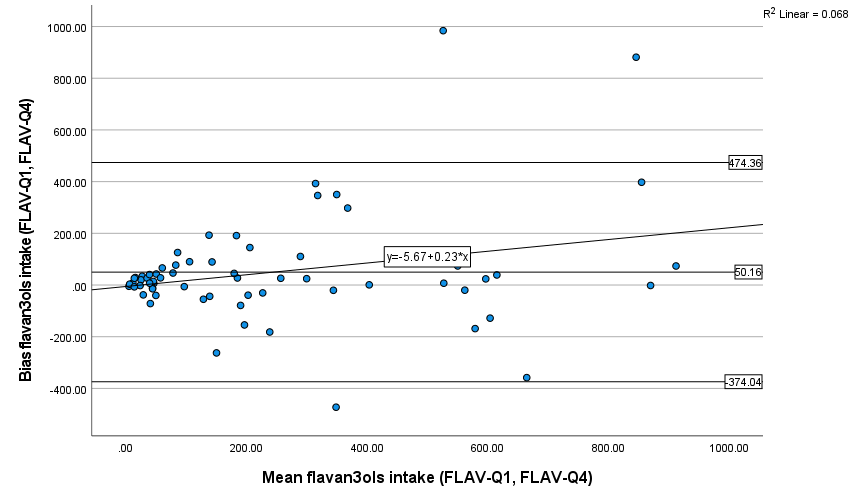 |
| 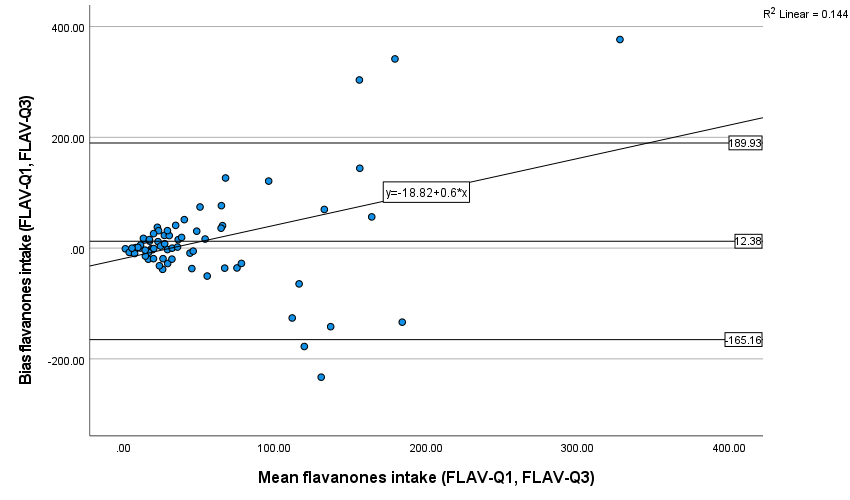 | 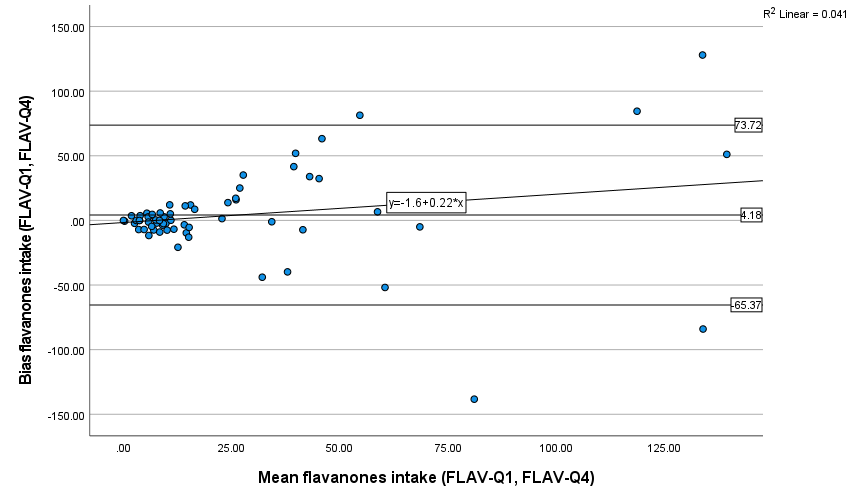 |
| 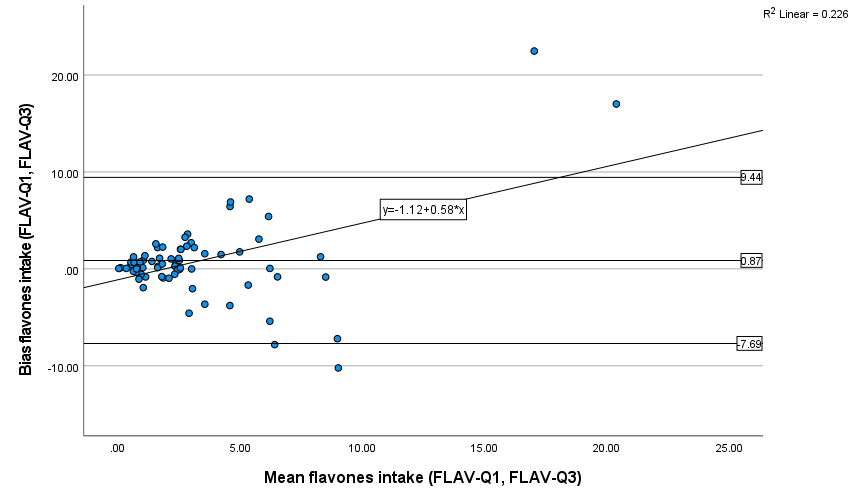 | 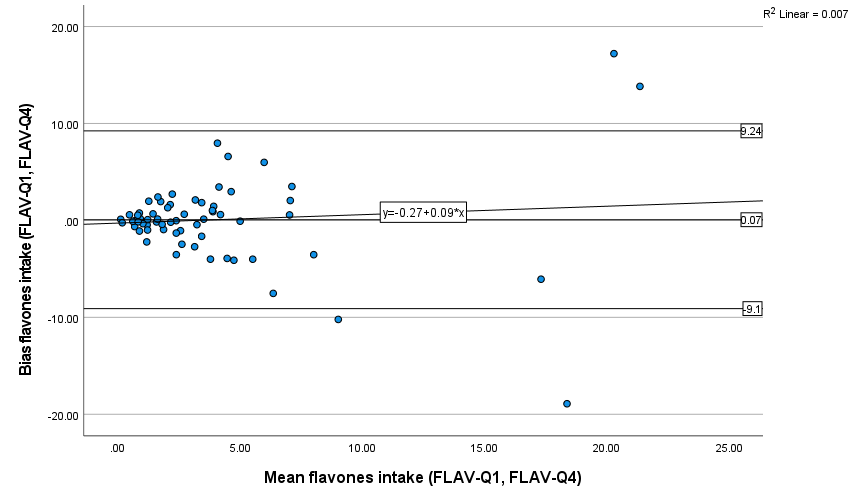 |

Fig.4 Bland-Altman plots (difference in intake (mg/day) (FLAV-Q1 –FLAV-Q3, FLAV-Q4) against the mean intake of flavonoids and subclasses (mg/day) [FLAV-Q1 + FLAV-Q3, FLAV-Q4)/2]) showing the relative validity of the FLAV-Q1 versus the FLAV-Q2, FLAV-Q3, FLAV-Q4 (A) flavonoids, (B) anthocyanin, (C) flavonol, (D) flavan3ol, (E) flavanone and (F) flavone intake.
